# MET Receptor Tyrosine Kinase Inhibition Reduces Interferon-Gamma (IFN-γ)-Stimulated PD-L1 Expression through the STAT3 Pathway in Melanoma Cells

**DOI:** 10.3390/cancers15133408

**Published:** 2023-06-29

**Authors:** Kyu Young Song, Yong Hwan Han, Heidi Roehrich, Mary E. Brown, Carlos Torres-Cabala, Alessio Giubellino

**Affiliations:** 1 Department of Laboratory Medicine and Pathology, University of Minnesota, Minneapolis, MN 55455, USA; 2 Masonic Cancer Center, University of Minnesota, Minneapolis, MN 55455, USA; 3 Microscopy and Cell Analysis Core, Mayo Clinic, Rochester, MN 55905, USA; 4 Department of Ophthalmology and Visual Neurosciences, University of Minnesota, Minneapolis, MN 55455, USA; 5 University Imaging Centers, University of Minnesota, Minneapolis, MN 55455, USA; 6 MD Anderson Cancer Center, The University of Texas, Houston, TX 77030, USA

**Keywords:** MET, PD-L1, melanoma, immunotherapy, tyrosine kinase inhibitors, STAT3

## Abstract

**Simple Summary:**

While the treatment of melanoma was revolutionized about a decade ago by the introduction of immunotherapies and targeted therapies, advanced melanoma remains a therapeutic challenge. Here we demonstrate a cross-talk between a checkpoint protein, PD-L1, and a receptor tyrosine kinase (RTK), MET. These findings open the possibility of combining selective inhibitors of these proteins to achieve synergistic efficacy in the treatment of melanoma.

**Abstract:**

Melanoma is the leading cause of death from cutaneous malignancy. While targeted therapy and immunotherapy with checkpoint inhibitors have significantly decreased the mortality rate of this disease, advanced melanoma remains a therapeutic challenge. Here, we confirmed that interferon-gamma (IFN-γ)-induced PD-L1 expression in melanoma cell lines. This increased expression was down-regulated by the reduction in phosphorylated STAT3 signaling via MET tyrosine kinase inhibitor treatment. Furthermore, immunoprecipitation and confocal immunofluorescence microscopy analysis reveals MET and PD-L1 protein–protein interaction and colocalization on the cell surface membrane of melanoma cells. Together, these findings demonstrate that the IFN-γ-induced PD-L1 expression in melanoma cells is negatively regulated by MET inhibition through the JAK/STAT3 signaling pathway and establish the colocalization and interaction between an RTK and a checkpoint protein in melanoma cells.

## 1. Introduction

Melanoma is the fifth most common malignant neoplasia in both men and women [1,2]. The incidence of melanoma is increasing slowly but steadily every year, but recent advances in treatment and patient management have resulted in improved survival [3]. Despite these advances, the prognosis and treatment of advanced disease remains a major challenge and new treatment approaches such as combined therapies are envisioned to pave the way to better manage these patients [4].

The *MET* proto-oncogene encodes for a member of the receptor tyrosine kinase (RTK) family, specifically the receptor for the hepatocyte growth factor/scatter factor (HGF/SF) [5]. Activation of the MET/HGF signaling pathway elicits a variety of cell signaling responses that result in a broad range of biological processes, such as cell proliferation, cell motility, and invasiveness [6], as well as regulation of the immune system [7]. Modulation of the immune response includes involvement in dendritic cell function [8] and neutrophilic antitumoral response [9] and this function has been postulated to be involved in the potential acquisition of resistance to immunotherapy [8]. Over the past three decades, a large body of evidence clearly demonstrated a central role of this protein in several malignancies [10,11], including malignant melanoma [12]. These studies validated MET as an attractive cancer biomarker [13] and therapeutic target [14]. However, the majority of the selective MET inhibitors that have been deployed in numerous clinical trials have resulted in little or no efficacy as a single agent. Thus, recent efforts explored the possibility to combine MET inhibition with other therapeutic options, with the hope of achieving synergistic effects.

Programmed death-ligand 1 (PD-L1) is expressed at the surface of tumor cells, tumor-associated macrophages, and T lymphocytes, and its expression can be induced by cytokines, such as interferons (IFNs) and tumor necrosis factors (TNFs) [15]. PD-L1 expression is mainly modulated in response to the release of IFN-γ by tumor-targeting immune cells [16]. PD-L1 expression on cancer cells inhibits PD-1-positive T-cells, a process known as adaptive immune resistance [17]. The expression of PD-L1 is mainly controlled by Janus kinase 1 (JAK1) and JAK2, the signal transducers and activators of transcription 1 (STAT1), and the interferon regulatory factor-1 (IRF1) pathway [18]. In various cell types, including cancer cells and myeloid cells, STAT3 can be induced as an atypical signal transducer of IFN-γ while the interplay between STAT1 and STAT3 determines the biological outcome [19]. STAT3 reportedly induces immunosuppression in cancer by upregulating PD-L1 and this overexpression of PD-L1 significantly associates with STAT3 phosphorylation [20]. Post-transcriptionally, PD-L1 expression can also be negatively regulated by several microRNAs [16].

The treatment of melanoma was revolutionized about a decade ago by the introduction of immunotherapies targeting checkpoint proteins [21]. Among them, the PD-L1 protein, expressed on melanoma cells, has emerged as a viable and effective target to disrupt the mechanism of cell evasions initiated by tumor cells [22,23]. This mechanism also represents an important form of resistance to other anti-tumor therapies. Therapeutic blockade of PD-L1 and its ligand, PD-1, with selective checkpoint inhibitors, demonstrated excellent results, initially in the treatment of advanced melanoma [24] and lately in a large number of other malignancies [21]. However, these therapies are effective in only a small percentage of patients, and some of the responders eventually relapse. Thus, there is an unmet need to find alternative therapeutic options for these patients [25].

We previously demonstrated that the protein expression, as assessed by immunohistochemistry, of MET and PD-L1 strongly correlated in advanced, metastatic melanoma [26]. In the present study, we further investigate their relationship by exploring the potential regulation of PD-L1 expression in melanoma by treatment with selective anti-MET tyrosine kinase inhibitors and the potential association between these two surface receptors.

## 2. Materials and Methods

### 2.1. Cell Culture and Treatments

The human melanoma cell lines (RPMI-7951, SH-4, and SK-MEL-28) were obtained directly from ATCC and were cultured following ATCC’s recommendations. Characteristics of these four cell lines are summarized in Table S1. The WM35 cell line was obtained from the Wistar Institute and cultured with tumor-specialized media containing 2% FBS.

Cells were treated with 25 ng/mL of IFN-γ (570202, BioLegend, San Diego, CA, USA) and MET inhibitors for 48 h with fresh replacements after 24 h. Cells were treated with MET-specific inhibitors (Crizotinib and PHA665752) purchased from Selleckchem (S1068 and S1070, Houston, TX, USA), at doses as specified in each experimental condition.

### 2.2. Flow Cytometry Analysis

After cells were treated as described in Section 2.1 and counted, 1 million cells per sample were re-suspended in 100 μL of staining buffer (phosphate-buffered saline plus 2% fetal bovine serum) and incubated with rat anti-c-MET FITC (11-8858-42, 5 μL/reaction, Invitrogen, Waltham, MA, USA) and mouse anti-CD274 BV 421 (329714, 5 μL/reaction, BioLegend) on ice for a minimum 30 min. After washing samples with staining buffer twice, data were acquired with a BD LSRII flow cytometer using BD FACSDiva software v8.0 (BD Bioscience, San Jose, CA, USA) and analyzed using Flowjo v10 (Flowjo, LLC, Ashland, OR, USA).

### 2.3. Western Blot Analysis

Harvested cells were washed twice with PBS at 4 °C, and 0.2 mL of lysis buffer (50 mM Tris-HCl [pH 7.5], 150 mM NaCl, 0.25% sodium deoxycholate, 0.1% Nonidet P-40, 0.1% Triton X-100, 50 mM NaF, 1 mM dithiothreitol, 0.5 mM phenylmethyl sulfonyl fluoride, 50 mM sodium pyrophosphate, 10 mM sodium vanadate, and 1X protease inhibitor cocktail [11836153001, Roche, Basel, Switzerland]) was added directly to the culture dishes. After centrifugation, the supernatant was transferred to a new tube and SDS-PAGE sample buffer was added to the supernatant. Approximately 30 μg of proteins from each lysate was resolved by SDS-PAGE using a 4–20% polyacrylamide gradient gel (4561094, Bio-Rad, Hercules, CA, USA). Gels were electroblotted onto polyvinylidene difluoride (PVDF) membranes (IPVH00010, Amersham Bioscience, Piscataway, NJ, USA) in transfer buffer (48 mM Tris-HCl, 39 mM glycine, and 20% methanol). Membranes were blocked in blocking solution (5% dry milk and 0.1% Tween 20 in Tris-buffered saline) overnight at 4 °C. Immunoblotting with MET (AF276, 0.5 μg/mL, R&D systems, Minneapolis, MN, USA), Phospho-MET (AF2480, 0.5 μg/mL, Tyr1234/1235 or AF3950, 0.5 μg/mL, Tyr1349, R&D systems), and AKT (9272, 1:2000), Phospho-AKT (9271, 1:1000), ERK1/2 (9102, 1:2000), Phospho-ERK1/2 (9101, 1:3000), FAK (3285, 1:1500), Phospho-FAK (3281, 1:1000), GSK-3β (9315, 1:1500), Phospho-GSK-3α/β (9331, 1:1000), PD-L1 (13684, 1:1000), STAT3 (9139, 1:2500), Phospho-STAT3 (9131, 1:1000), and β-actin (3700, 1:2000) (Cell Signaling Technology, Danvers, MA, USA) was performed according to the manufacturer’s instructions. Signals were detected using ECL substrate (32209, ThermoFisher, Waltham, MA, USA), according to the manufacturer’s instructions. Western blot bands were analyzed with the Image Studio^TM^ Lite software (LI-COR, Lincoln, NE, USA).

### 2.4. Immunoprecipitation (IP)

Immunoprecipitation procedures were performed with protein G magnetic beads from Cell Signaling (Cat. #70024). All the cell lysates were pre-cleared with lysate buffers and 500 μg of cell lysates were used for each immunoprecipitation. MET (AF276, 5 ug/reaction, R&D systems) and PD-L1 (13684, 1:60) antibodies were allowed to bind overnight at 4 °C, then 20 μL of pre-washed Protein G magnetic beads were added. Beads were washed five times in lysis buffer, then resuspended in 40 μL of 2X sample buffer. Supernatants with the immunoprecipitants were separated on 4–20% SDS-PAGE gels, as previously described. A monoclonal IgG was also used as a control for the immunoprecipitation (Figure S3).

### 2.5. Immunofluorescence Assay (IF)

To characterize and quantify the expression and colocalization of MET and PD-L1, four melanoma cell lines (RPM1-7971, SK-MEL-28, SH-4, and WM35) were platted on 8 well chamber slides. These cells were paraformaldehyde-fixed and foremost stained with wheat germ agglutinin, conjugated to AlexaFluor 555 (W32464, Invitrogen), following the manufacturer’s instructions. After permeabilization of cell membranes with 0.2% Triton-X 100 and blocking with 10% normal donkey serum, the cell lines were double-stained overnight at 4 °C with MET (#8198, 1:200, Cell Signaling, Danvers, MA, USA) and PD-L1 (ab210931, 1:500, Abcam, Waltham, MA, USA). The reaction was visualized using Hoechst and corresponding secondary antibodies. Secondary antibody staining alone was used as a negative control.

### 2.6. Confocal Imaging

Images were acquired on an Olympus BX2 upright confocal microscope equipped with a Fluoview 1000 confocal scan head and a UPlanApo N 60X 1.42 NA oil objective. The confocal aperture was set to 80 µm. The system was controlled by Olympus FluoView FV1000 software, version 4.1.1.5. DAPI was excited with a 405 nm, 25 mW solid-state diode laser (20% laser power), and emission was collected at 430–470 nm with a photomultiplier tube (PMT) gain of 435 V and an offset of 7. AlexaFluor488 was excited with a 488 nm, 30 mW Argon laser (10% laser power), and emission was collected at 505–525 nm with a PMT gain of 440 V and an offset of 6. AlexaFluor555 was excited with a 543 nm, 1 mW HeNe laser (85% laser power), and emission was collected at 560–640 nm with a PMT gain of 690 V and an offset of 7. AlexaFluor647 Plus was excited with a 635 nm, 10 mW HeNe laser (85% laser power), and emission was collected at 655–755 nm with a PMT gain of 705 V and an offset of 3. Twelve-bit 800 × 800 pixel images with a voxel size of 94 × 94 × 330 nm were collected.

### 2.7. Image Analysis

All image processing and analyses were performed with Imaris software, version 9.9.1 (Oxford Instruments, Carteret, NJ, USA). Images were background-subtracted by first applying a Gaussian filter with a filter width of 25 µm to a duplicate image followed by a subtraction of this filtered image from the original image. Next, a 3 × 3 × 3 median filter was applied and colocalization analysis commenced. The colocalization volume analyzed was bounded by a mask of the wheat germ agglutinin (WGA)-AlexaFluor555 image with the threshold set to an intensity value of 50. Colocalization was assessed in the AlexaFluor488 and AlexaFluor647 Plus channels and thresholds were automatically set by Imaris software using the method established by Costes SV, et al. [27], wherein the 2D histogram of the green and far-red channels is used and, starting with the highest intensity values, correlation coefficients are calculated at each threshold level until the correlation coefficient reaches zero. At this point, the threshold intensities for both channels are set. The software generates a colocalization channel that presents the colocalized voxels and contains the Pearson’s correlation coefficient for the colocalized volume. Pearson’s correlation coefficients were averaged, and standard deviations were calculated and presented in the immunofluorescence analysis data.

### 2.8. Statistical Analysis

All data are presented as the mean ± standard deviation (SD), and differences between the means were examined by student’s *t*-test or one-way ANOVA using the Newman–Keuls test in GraphPad Prism 5 (GraphPad Software, Boston, MA, USA). Differences with a value of *p* < 0.05 were considered statistically significant. At least three independent experiments were performed.

## 3. Results

### 3.1. MET Inhibitors Reduce IFN-γ-Induced PD-L1 Expression in Melanoma Cell Lines

**Figure 1:**
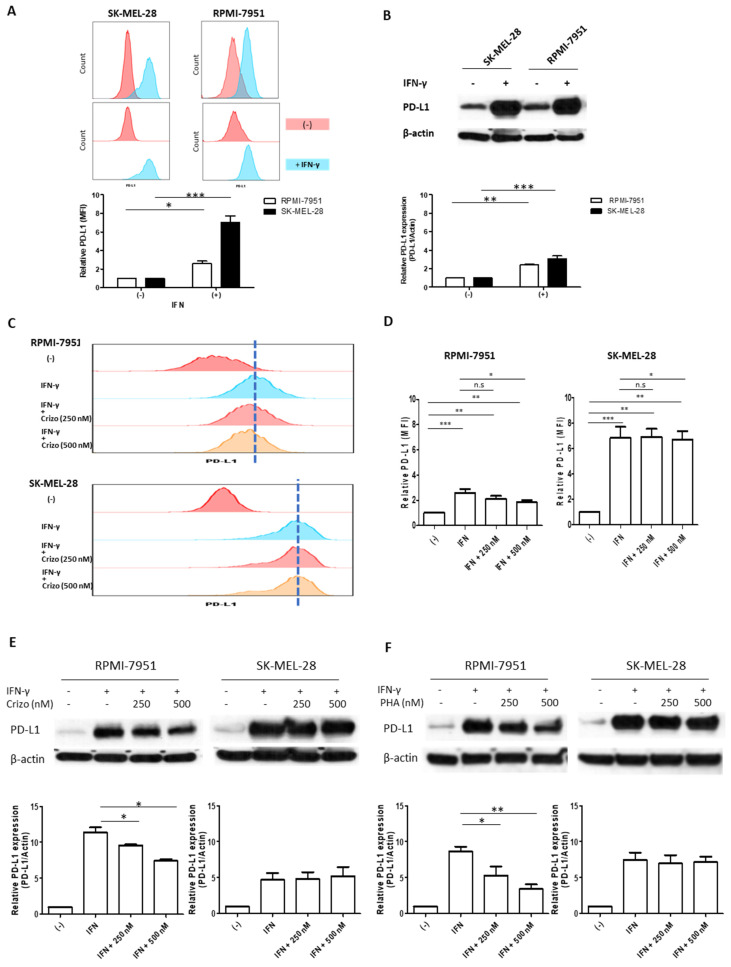
Interferon-γ treatment upregulates PD-L1 expression in melanoma cells, and MET inhibitors down-regulate interferon-γ induced PD-L1 expression in the melanoma cell lines. (**A**) Flow cytometry analysis of PD-L1 expression on the cell membrane of melanoma cells, treated with interferon-γ. Mean fluorescent intensity (MFI) values in the graph are mean ± SD calculated from three independent experiments. (**B**) Western blot analysis of PD-L1 expression in total protein extracts from melanoma cells. All experiments were treated with interferon-γ (50 ng/mL) for 48 h. (**C**) Flow cytometry analysis from one independent experiment showing the labeled cells in the absence (−) or presence (+) of IFN-γ with the indicated concentration of MET inhibitor (Crizotinib). (**D**) Flow cytometry analysis of PD-L1 protein on the cell membrane of melanoma cells upon 48 h treatment with IFN-γ alone or in combination with the MET inhibitors as indicated concentration. Western blot analysis of PD-L1 expression on total protein extracts from melanoma cells treated with 48 h with IFN-γ alone or in combination with Crizotinib (**E**) or PHA665752 (**F**). * *p* < 0.05, ** *p* < 0.01, *** *p* < 0.005. The uncropped blots are shown in Figure S4.

We first wanted to verify the induction of PD-L1 expression in our melanoma cells by IFN-γ, the main known stimulator of the PD-L1 protein. The RPMI-7951 and SK-MEL-28 melanoma cell lines express a baseline level of PD-L1, as detected by flow cytometric analysis (Figure 1A, upper panel), expressed as the mean fluorescence intensity (MFI) value. When these cells are treated with IFN-γ for 48 h, there is a clear induction of PD-L1 expression in both cell lines (Figure 1A, middle panel); quantitative analysis, represented by the bar graph of Figure 1A, demonstrates the statistical significant increase in PD-L1 expression after IFN-γ. Similar results are also confirmed by Western blot protein analysis (Figure 1B) with statistical significance, as shown in the bar graph.

**Figure 2:**
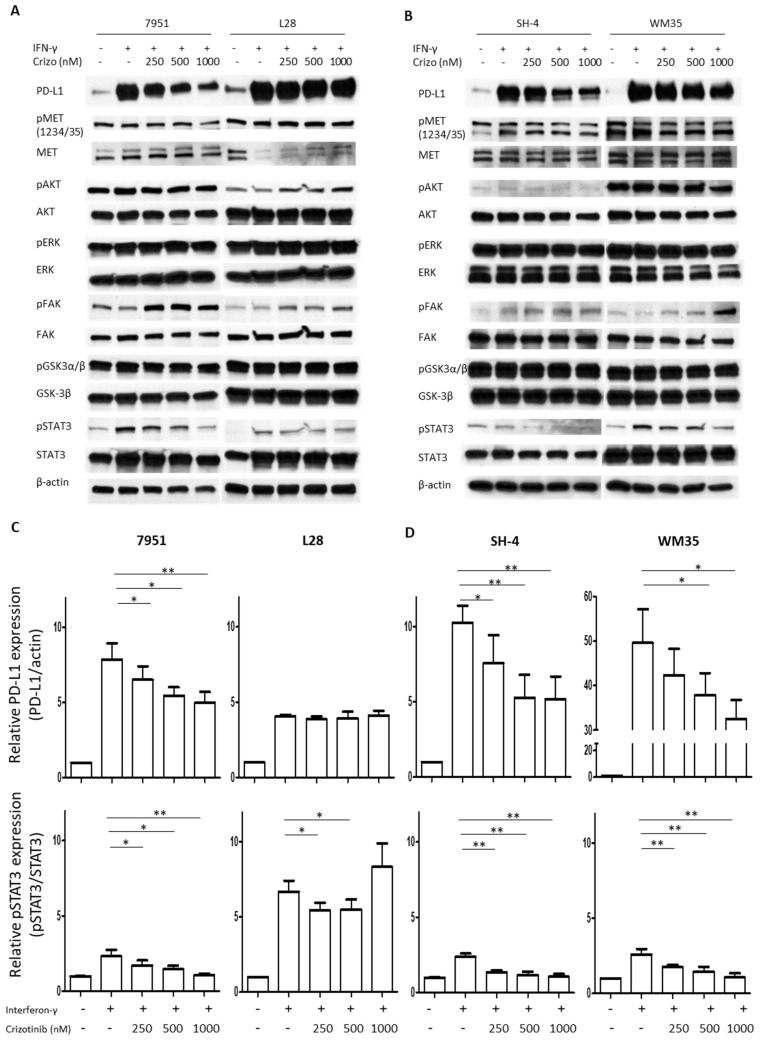
MET inhibition diminishes IFN-γ-induced PD-L1 expression by reducing STAT3 activation. Analysis of MET, AKT, ERK, FAK, GSK3β, and STAT3 activation/expression together with PD-L1 expression in lysates obtained from four melanoma cells treated for 48 h with IFN-γ (25 ng/mL) alone or in combination with the Crizotinib (Crizo) (**A**,**B**). Densitometric quantification for PD-L1 (**C**) and phosphorylated-STAT3 expression (**D**) were quantified using Image Studio^TM^ Lite v 5.2 (LI-COR, USA) software and quantitative data are presented as mean ± SD based on 3 independent experiments. * *p* < 0.05, ** *p* < 0.01. The uncropped blots are shown in Figure S5A,B.

To explore whether MET-targeted selective therapy can interfere with the modulation of PD-L1 expression induced by INF-γ in melanoma cells, we performed flow cytometry experiments on Crizotinib-treated cells. This experiment shows that the selective MET inhibitor affects PD-L1 expression levels in the RPMI-7951 cell line in a dose-dependent manner (Figure 1C,D) and, to a more limited extent, in SK-MEL-28 cells. We wanted to confirm these data by Western blot analysis. For this experiment we used two different MET inhibitors, Crizotinib (Figure 1E) and PHA 665752 (Figure 1F); our data show that both inhibitors have a dose-dependent effect in lowering PD-L1 expression in the RPMI cell lines, but no significant effect in the SK-MEL-28 cell lines, thus, supporting our flow cytometry data. Next, we expanded our analysis in two additional melanoma cell lines. As shown in Figure 2, MET inhibition with Crizotinib is also effective, in a dose-dependent manner, in reducing PD-L1 expression in the melanoma SH4 and WM35 cell lines. Thus, selective MET inhibition is able to reduce the expression of PD-L1 in three out four melanoma cell lines.

### 3.2. MET Inhibitors Affect PD-L1 Expression through STAT3 Signaling Pathways

To explore the potential mechanism of cross-talk between MET and PD-L1, we investigated several downstream signaling effectors of these two proteins. Four melanoma cell lines were treated with INF-γ, to induce the expression of PD-L1, and increasing concentrations of the MET-inhibitor Crizotinib were added, from 250 nM up to 1 μM. As described above, inhibition of PD-L1 expression is achieved in a dose dependent manner in three of the four cell lines. Crizotinib also decreases MET phosphorylation in these three cell lines, and, to an apparently lesser extent, in the SK-MEL-28 cell line. Interestingly, the MET inhibitor reduces, in a dose-dependent manner, the phosphorylation of STAT3, while no effect is detected in the total expression of this protein (Figure 2). There are also no effects on the examined AKT, ERK, FAK, and GSK-3β proteins and their activation through phosphorylation.

To further validate the dependence on STAT3 phosphorylation, we used a selective STAT3 small molecule inhibitor (BP-1-102) in melanoma cells. In this experiment, PD-L1 expression is decreased when STAT3 phosphorylation is inhibited (Figure S2), suggesting that the upregulation of PD-L1 expression induced by INF-γ depends on STAT3 phosphorylation.

### 3.3. Interaction and Colocalization of MET and PD-L1

**Figure 3:**
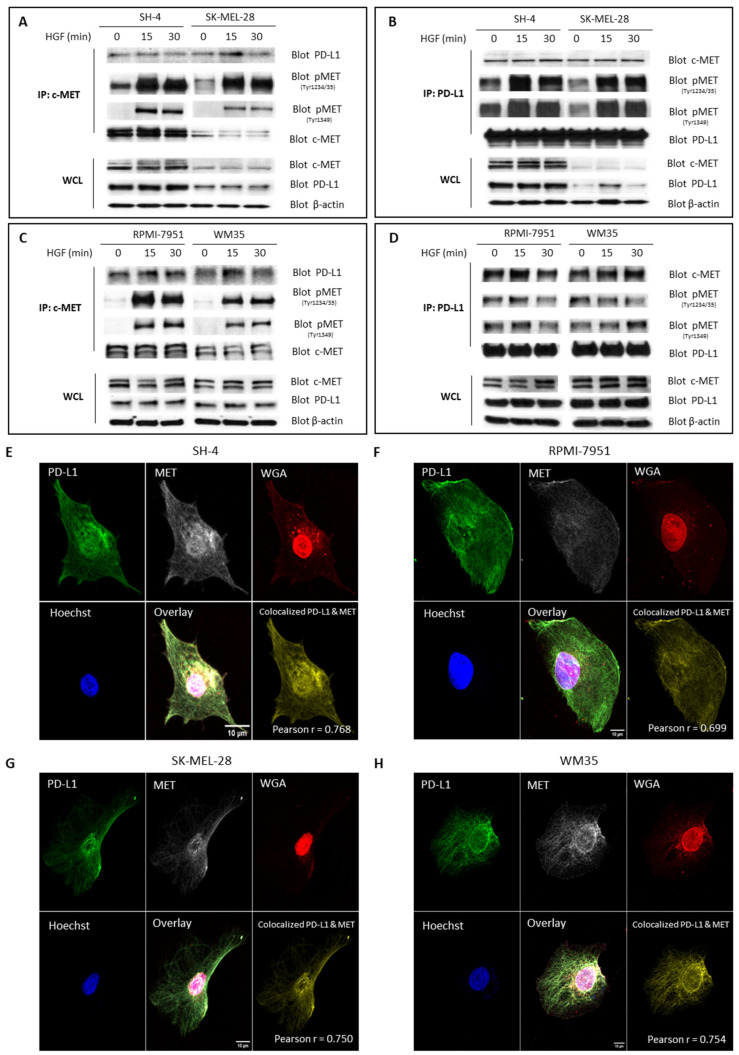
Co-immunoprecipitation of MET and PD-L1 from lysates of melanoma cells. Extracts were prepared from each cell line after treatment with HGF (25 ng/mL) at the indicated times and immunoprecipitated using c-MET (**A**,**C**) or PD-L1 (**B**,**D**) antibodies. Each immunoprecipitant was probed for the presence of MET, pMET (Tyr 1234/35), pMET (Tyr 1349), and PD-L1. Immunoblotting of whole cell lysates (WCL) was performed with antibodies against c-MET and PD-L1 to examine total levels of MET and PD-L1. WCL immunoblotting with actin was used as the loading control. Co-localization analysis of MET and PD-L1 expression by immunofluorescence analysis. Colocalization of PD-L1 (Alexa Fluor 488) with MET (Alexa Fluor 647) in the plasma membrane (WGA, Alexa Fluor 555) as analyzed by confocal laser scanning microscopy in SH-4 (**E**), RPMI-7951 (**F**), SK-MEL-28 (**G**), and WM-35 (**H**) melanoma cell lines. Pearson’s correlation between PD-L1 and MET expression in melanoma cells is shown in the colocalized PD-L1 and MET column in each cell line. Scale bars: 10 μm. The uncropped blots are shown in Figure S6A–D.

The interference of a selective MET inhibitor in the expression of PD-L1 raises the possibility of a coupling of these two receptors in a complex on the cell surface. To test this hypothesis, we performed co-immunoprecipitation experiments. We used the same melanoma cell lines described above, expressing endogenous and physiological levels of both proteins. The lysates of these cell lines, treated with recombinant HGF, were subjected to immunoprecipitation with a MET-selective antibody; when we performed Western blot analysis, we were able to demonstrate the presence of PD-L1 in the immunoprecipitates, demonstrating a physical interaction between the two receptors (Figure 3A,C). We confirmed these results by performing the reverse experiment, in which the immunoprecipitates treated with the PD-L1 specific antibody were immunoblotted for MET (Figure 3B,D).

These results demonstrate that endogenous MET interacts with PD-L1 in melanoma cells. To further investigate the interaction between these two receptors, we employed immunofluorescent staining (Figure 3E–H) to visualize the colocalization of these two receptors. Staining with MET (AF647), PD-L1 (AF488) and wheat germ agglutinin (WGA-AF555), a plasma membrane tracker, demonstrates that MET and PD-L1 co-localize on the cell membranes. To quantify the colocalization of MET and PD-L1 fluorescence signals, we calculated the Pearson’s correlation coefficient (PCC) in both channels for cells imaged independently. Colocalization analysis of MET and PD-L1 on the four melanoma cell lines reveals a PCC of 0.768 (±0.04) for the SH-4 cell line; 0.699 (±0.04) for the RPMI-7951 cell line; 0.750 (±0.09) for the SK-MEL-28 cell line; and 0.754 (±0.03) for the WM35 cell line. Taken together, these data demonstrate a physical interaction of MET and PD-L1 on the cell surface of melanoma cell lines.

## 4. Discussion

Dysregulation of receptor tyrosine kinases (RTKs) signaling represents a major hallmark of a wide range of human malignancies. Several RTK inhibitors have been developed, some of which have demonstrated some degree of efficacy in selected tumors. However, current tyrosine kinase inhibitors (TKIs) of these receptors have, in general, little utility as single agents in the treatment of melanoma. For example, KIT inhibitors demonstrate some, but limited, utility for acral melanoma [28].

Among the RTKs family of receptors, MET has been extensively studied. Aberrant activation of the MET/HGF pathway has been found in many types of cancer [5] and it is associated with disease progression, metastasis, and drug resistance [11,29,30,31,32]. MET is also involved in the regulation of the immune system. Among these activities, MET phosphorylation can activate inflammatory pathways through the signal transducer and activator of transcription (STAT) and nuclear-factor-kB (NF-kB) [7].

The immune system plays a pivotal role in the tumor microenvironment [33]. The discovery and targeting of checkpoint proteins and their role in tumor immune evasion has opened a great opportunity in oncology. The expression of PD-L1 induced by IFN-γ is a significant mechanism for tumors to evade the immune system [16]. Tumor cells use PD-L1 to disrupt T-cell-mediated immune surveillance and PD-L1 expression in tumor cells is associated with poor clinical outcomes [34,35]. In particular, patients with PD-L1-overexpressing melanoma have a 44–51% response rate to anti-PD-L1-directed therapy. In contrast, patients with PD-L1-negative melanoma have a response rates of about only 6–17% of cases [35]. PD-L1 also plays other roles in regulating the biological functions of cancer cells, including involvement in an epithelial-to-mesenchymal transition (EMT) phenotype [36,37], which contributes to their malignant behavior. These functions may represent an important point of connection with RTK signaling.

We have previously reported that the expression of MET and PD-L1 appear to positively correlate in metastatic melanomas, while this correlation is absent in primary melanoma and benign melanocytic nevi [26]. Similar studies correlating the expression of these two proteins have been previously reported [38,39].

Our study shows that inhibition of MET by selective inhibitors results in a dose-dependent decrease in IFN-γ-induced PD-L1 expression in melanoma cells. Furthermore, a STAT3-specific inhibitor, BP-1-102, is able to decrease the expression of PD-L1 in a dose-dependent manner in melanoma cells. The induction of PD-L1 expression on tumor cells by IFN-γ, a type II interferon, is an important mechanism adopted by melanoma to evade immunosurveillance [40,41]. Thus, our study demonstrates a cross-talk between the MET and IFN-γ signaling pathways, an interesting finding that may have therapeutic implications. MET inhibition downregulates PD-L1 expression via the inactivation of STAT3. Our results show that the expression of PD-L1 induced by IFN-γ is regulated through the JAK/STAT pathway in a dose-dependent manner with MET inhibition. This finding is in accordance with a study by Song et al. reporting that dysregulation of the JAK/STAT pathway may cause oncogenic PD-L1 overexpression [42]. Our results indicate that the MET inhibitor Crizotinib significantly reduces STAT3 phosphorylation in a concentration-dependent manner but has no effect on total STAT3. Other important downstream proteins, such as GSK-3, PI3K-AKT, and RAS-ERK, appear to be unaffected. IFN-γ secreted by immune cells activates JAK/STAT signaling to induce PD-L1 in tumor cells and various immune cells, including macrophages, T-cells, B-cells, and dendritic cells. IFN-γ-induced PD-L1 expression in tumor cells drives tumor progression and makes tumors dependent on PD-1/PD-L1-mediated T-cell inhibition for survival. Therefore, inflamed tumors exhibiting an IFN-γ signature are more responsive to anti-PD-1 therapy [43]. In contrast to anti-PD-1 therapy, which blocks T-cell inhibition and reactivates T-cells, MET inhibitors act by inhibiting IFN-γ-induced JAK–STAT signaling and its downstream PD-L1 expression in human melanoma cells. This helps to protect against T-cell exhaustion and enhance their immune responses. The opposing effects of these mechanisms can explain their differential impacts on JAK–STAT signaling and PD-L1 expression in tumor cells.

These results demonstrate that MET inhibitors, besides modulating the direct intended target, may have additional indirect effects on other signaling pathways and that it may be beneficial to add these inhibitors to other therapies, with the possibility of providing additional therapeutic benefits. It was interesting to observe that the effect of the MET inhibitors was variable across the melanoma cell lines, suggesting a complex mode of regulation, which may be, only in part, explained by the different expressions of these two proteins in different cell lines, as well as in human samples, as we have previously reported [26]. The four cell lines used in this study were those expressing high levels of MET and PD-L1 out of a total of 18 melanoma cell lines (Figure S1). As shown in Figure S1, expression of PD-L1 in SK-MEL-28 is about five times lower than that in RPMI-7951 cells, where PD-L1 is most highly expressed. Interestingly, upon interferon treatment, membrane and total PD-L1 expression is significantly higher in SK-MEL-28 cells than in RPMI-7951 (Figure 1A,B). Thus, the level of PD-L1 itself appears to modulate the inhibitory effect of Met inhibitors on PD-L1, a consideration that may have therapeutic implications. Another potential explanation is that activation of alternative signaling pathways may, in part, explain these results; indeed, the melanoma cell line that lacked an apparent response to MET inhibition, SK-MEL-28, has, for example, an EGFR mutation (see Supplemental Table S1) that may have contributed to this phenomenon. Additional studies are necessary to fully understand the mechanism behind this observation.

**Figure 4:**
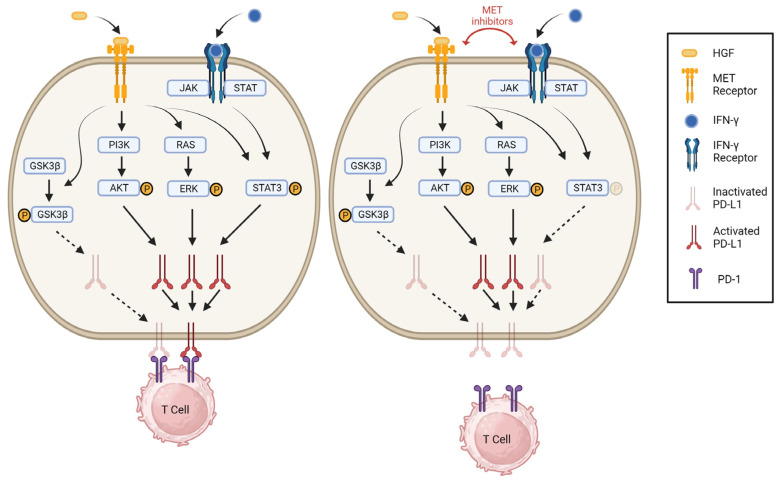
Schematic diagram of the proposed working model for the inhibition of PD-L1 by MET inhibitors in melanoma cell lines. MET inhibitors inhibit IFN-γ-induced STAT3 phosphorylation, leading to reduced PD-L1 expression and membrane presentation. IFN-γ, interferon-gamma (Created with BioRender.com: OD25HC9TFN).

In the present study, we also demonstrate a physical interaction between MET and the PD-1 receptor PD-L1 on the surface of melanoma cells. We demonstrate this interaction with two methodologies, co-immunoprecipitation and colocalization analysis. We performed co-immunoprecipitation studies, which demonstrate that MET and PD-L1 interact (Figure 3). To corroborate this finding, we also performed colocalization studies using fluorescent-labeled antibodies and a cell membrane marker; these immunofluorescence data show that MET and PD-L1 localize in close proximity on the surface cell membrane of melanoma cells. Our proposed working model for PD-L1/MET interaction is illustrated in Figure 4**.**

MET has been demonstrated to interact with a growing number of surface cellular proteins, shedding light on the complexity of cross-talks on the cell membrane and downstream signaling pathways [32]. These interactions, while usually not essential for the survival of the cell, allow for the fine modulation of the signals derived from the surrounding microenvironment. Surface receptors interacting with MET include proteins such as B plexin family members [44,45], other receptor tyrosine kinases, such as EGFR [46], the transmembrane receptor for hyaluronic acid CD44 [47,48], integrins [49], and FAS [50]. All these interactions contribute to the amplification of MET signaling and the modulation of several biological processes, such as regulation of cell adhesion, pro-invasive signaling, and modulation of apoptotic pathways.

## 5. Conclusions

In conclusion, our study provides new insights into the interaction between RTKs and checkpoint proteins and opens the possibility of combining selective inhibitors of these proteins to achieve a synergistic effect in preventing T-cell exhaustion and to enhance the immune response in melanoma treatment.

## Data Availability

Data are available upon request.

## Supplementary Materials

The following supporting information can be downloaded at: https://www.mdpi.com/article/10.3390/cancers15133408/s1, Figure S1: Western blot analysis of MET and PD-L1 in 18 human melanoma cell lines. Each bar in the graph represents results of densitometric analysis; error bars represent SD of triplicates. Intensities were analyzed by Image Studio^TM^ Lite 5.2 software. Figure S2: Inhibition of STAT3 downregulates PD-L1 expression in melanoma cell lines. Figure S3: MET and PD-L1 co-immunoprecipitation; immunoglobulin control. Figure S4: Uncropped western blots in the Figure 1. Figure S5A,B: Uncropped western blots in the Figure 2. Figure S6A–D: Uncropped western blots in the Figure 3. Table S1: Characteristics of the four melanoma cell lines used in our experiments.
