# Supplementary material for: MET Receptor Tyrosine Kinase Inhibition Reduces Interferon-Gamma (IFN-γ)-Stimulated PD-L1 Expression through the STAT3 Pathway in Melanoma Cells
Source: Cancers (Basel). 2023 Jun 29;15(13):3408. doi: 10.3390/cancers15133408 (PMC10340457; doi:10.3390/cancers15133408)
Supplement: Supplementary file 1 [file cancers-15-03408-s001.zip › Supplemental Figure S1.pdf]

Supplemental Figure S1

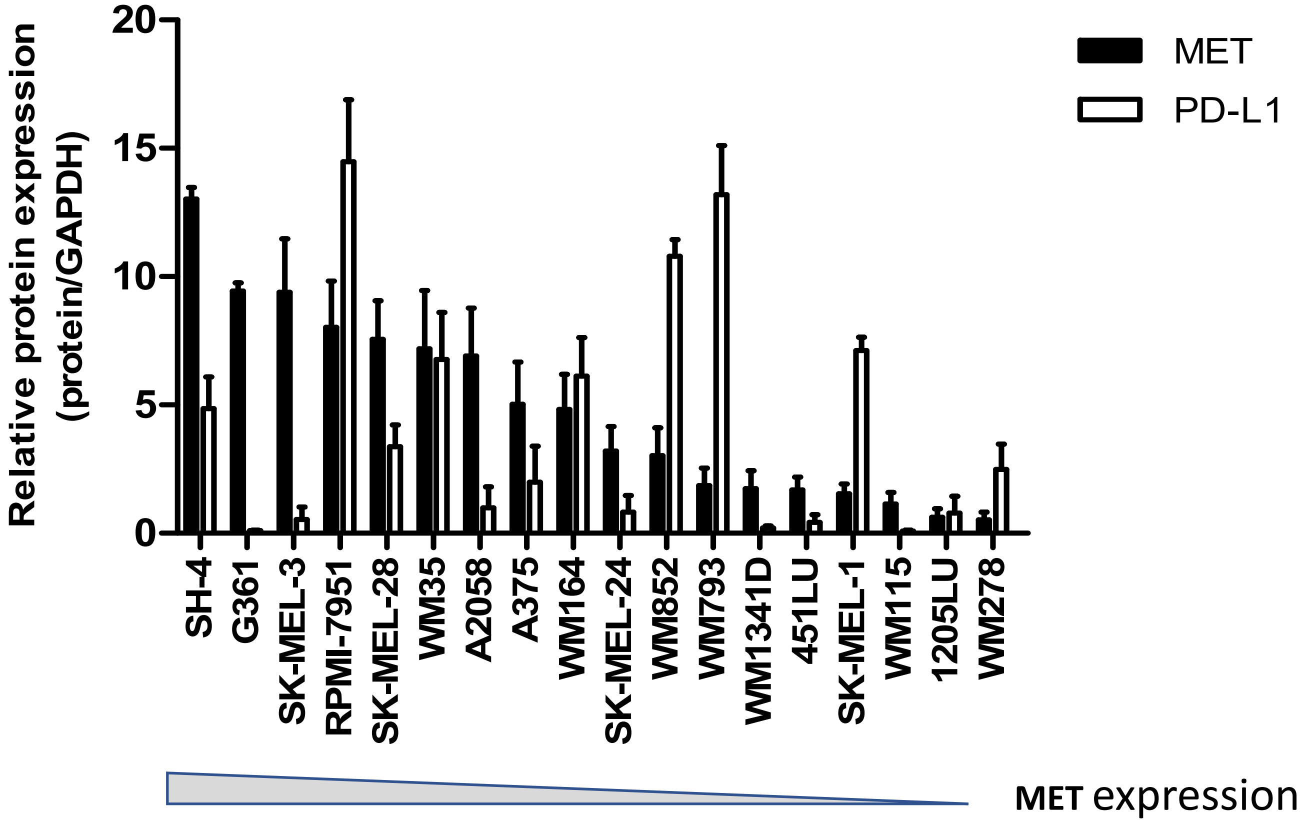

Supplemental Figure 2

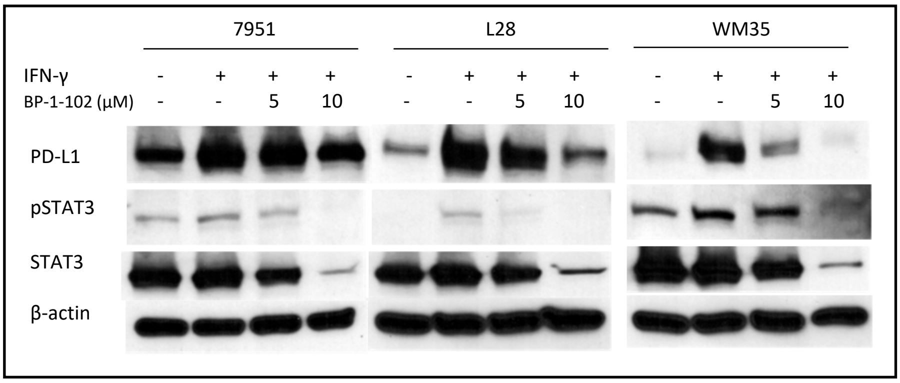

Supplemental Figure 3

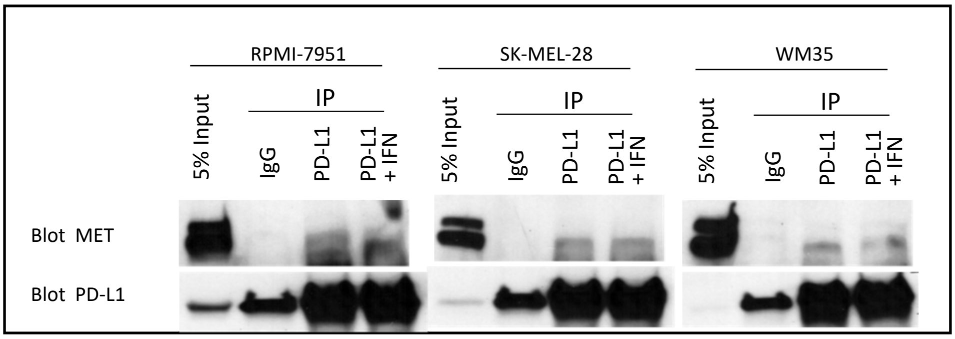

Supplemental Table 1

Name	Tumor source	Tissue	STR profile	Sequence variations	Pigmentation
**WM35**	Primary	superficial spreading	Link	BRAF p.Val600Glu (c.1799T>A)	-
**SK-MEL-28**	Metastasis	Axillary Lymph node	Link	BRAF p.Val600Glu (c.1799T>A)<br>CDK4 p.Arg24Cys (c.70C>T)<br>EGFR p.Pro753Ser (c.2257C>T)<br>PTEN p.Thr167Ala (c.499A>G)<br>TP53 p.Leu145Arg (c.434T>G)	-
**RPMI-7951**	Metastasis	Lymph node	Link	BRAF p.Val600Glu (c.1799T>A)<br>PTEN deletion	-
**SH-4**	Metastasis	Pleural effusion	Link	BRAF p.Val600Glu (c.1799T>A)	Yes
