# Supplementary material for: MET Receptor Tyrosine Kinase Inhibition Reduces Interferon-Gamma (IFN-γ)-Stimulated PD-L1 Expression through the STAT3 Pathway in Melanoma Cells
Source: Cancers (Basel). 2023 Jun 29;15(13):3408. doi: 10.3390/cancers15133408 (PMC10340457; doi:10.3390/cancers15133408)
Supplement: Supplementary file 1 [file cancers-15-03408-s001.zip › Supplemental Figure S4.pdf]

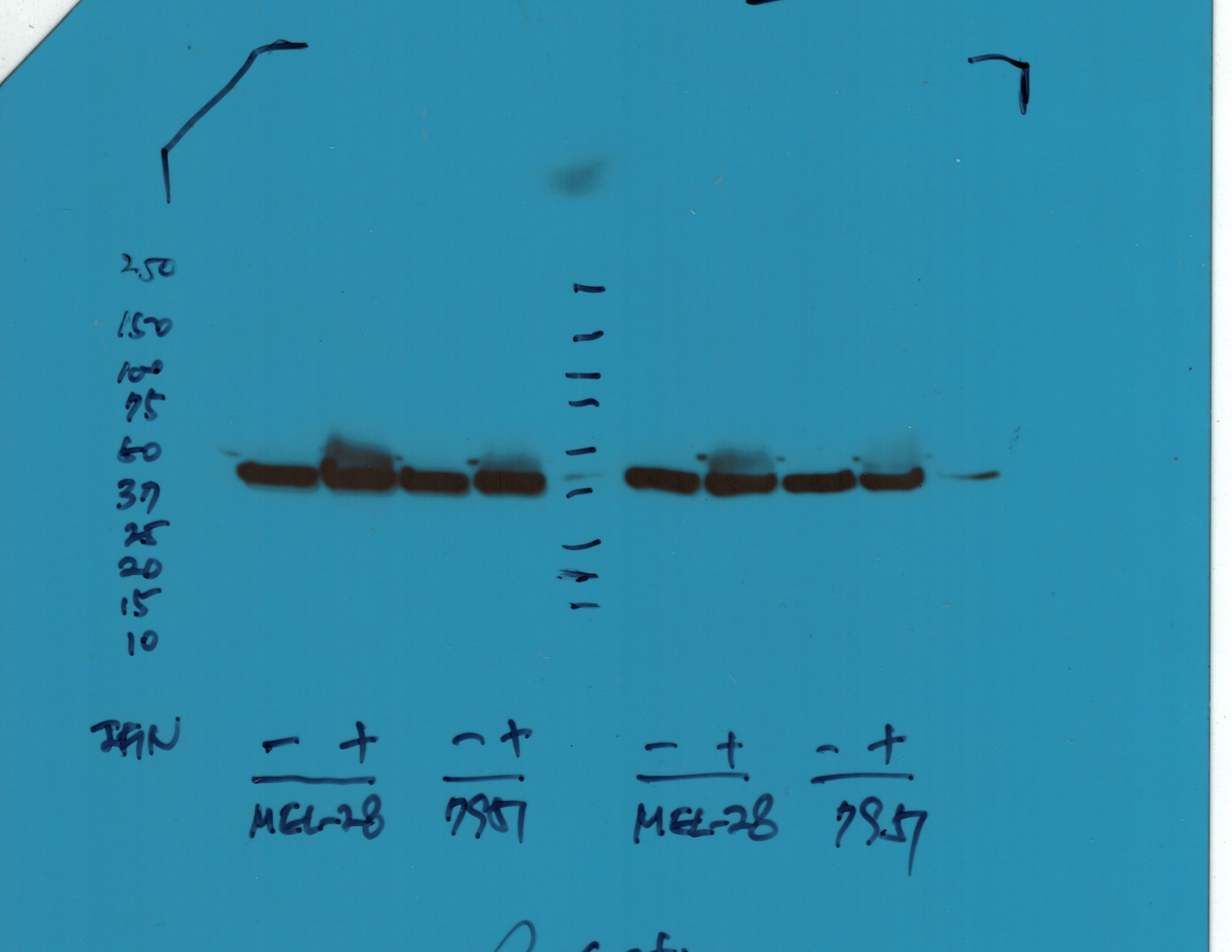
250
150
100
75
50
37
25
20
15
10
IFN
− + − + − + − +
MEL-28
MEL-28

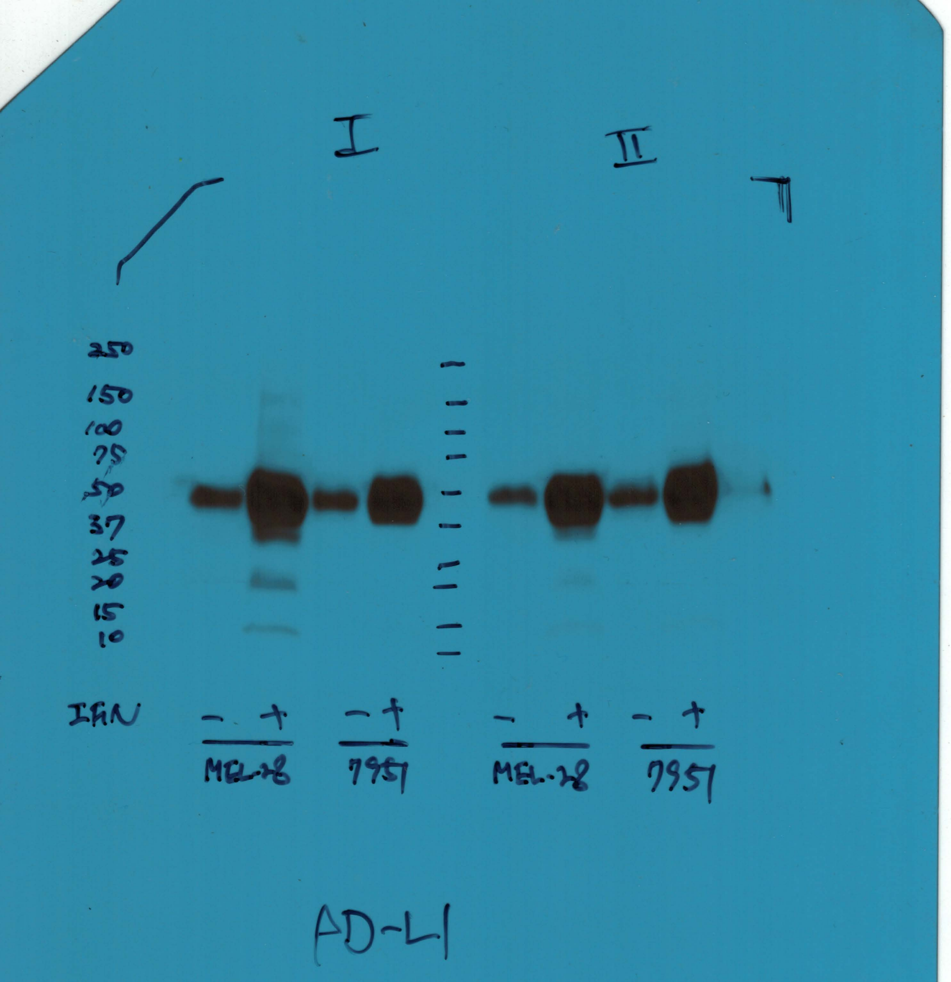
I
II
250
150
100
75
50
37
25
20
15
10
IFN
− + − + − + − +
MEL-28
7951
MEL-28
7951
PD-L1

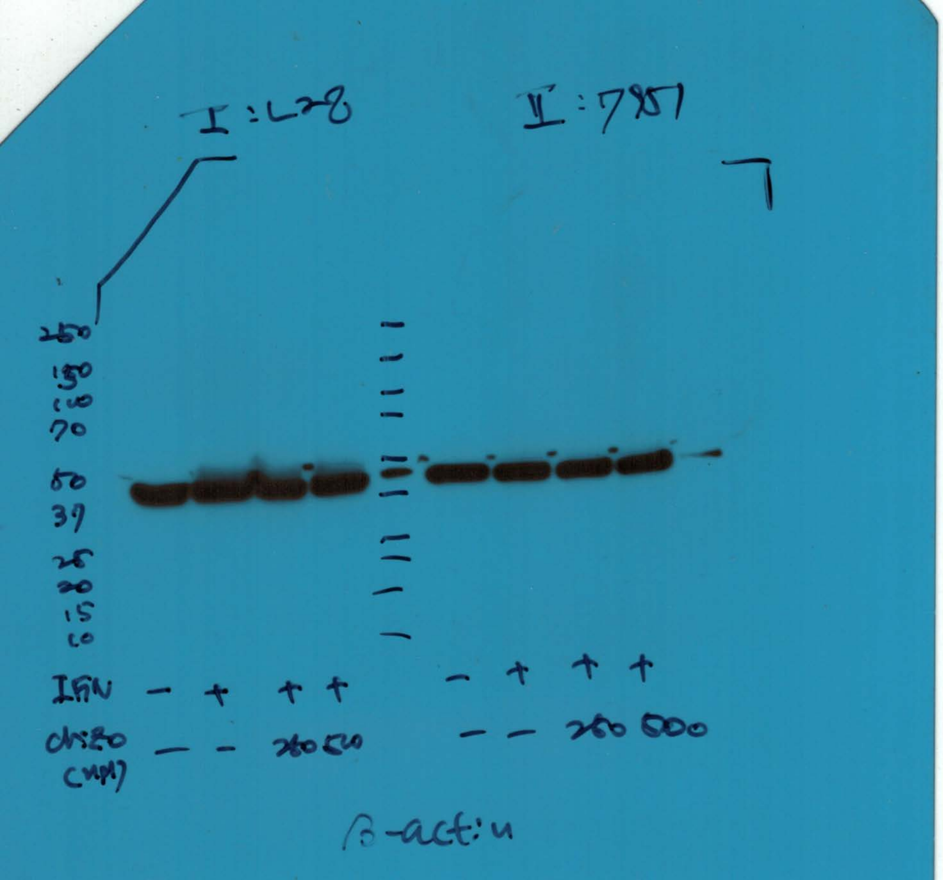
I : L28
II : 7851
250
150
100
70
50
37
25
20
15
10
IFN − + + +
− + + +
− − 250 500
− − 250 500
(nM)
β-actin

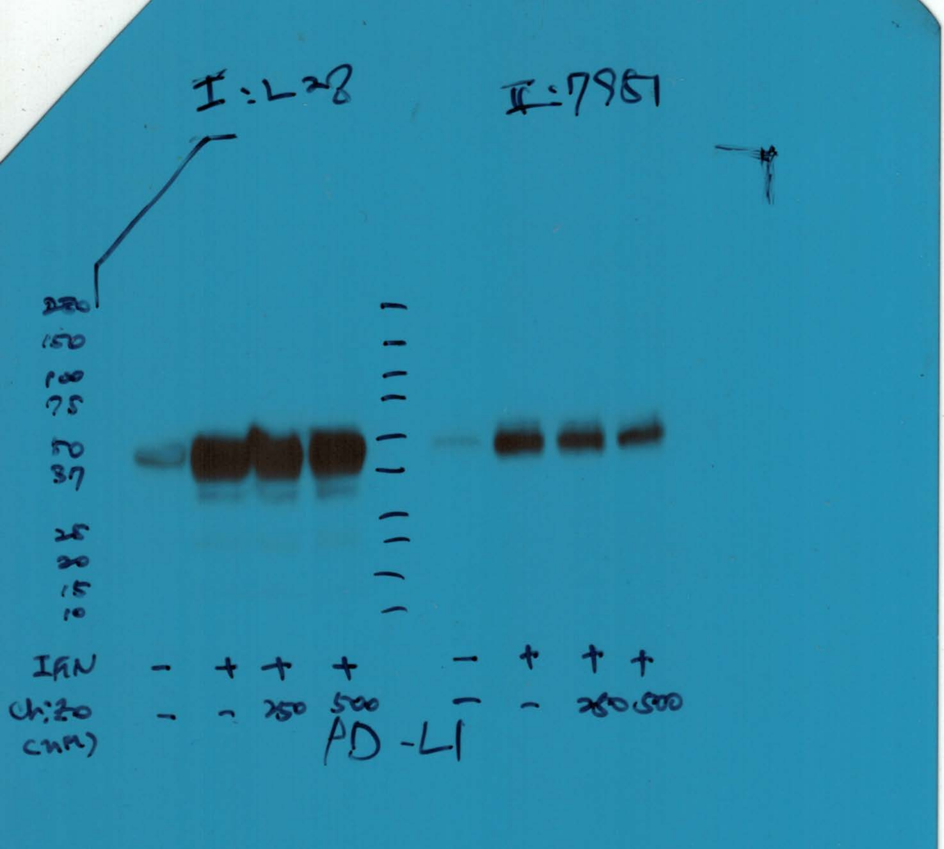

I : L28
II : 7951
250
150
100
75
50
37
25
20
15
10
IFN - + + + - + + +
Ch:20 - - 250 500 - - 250 500
(nM)
PD-L1

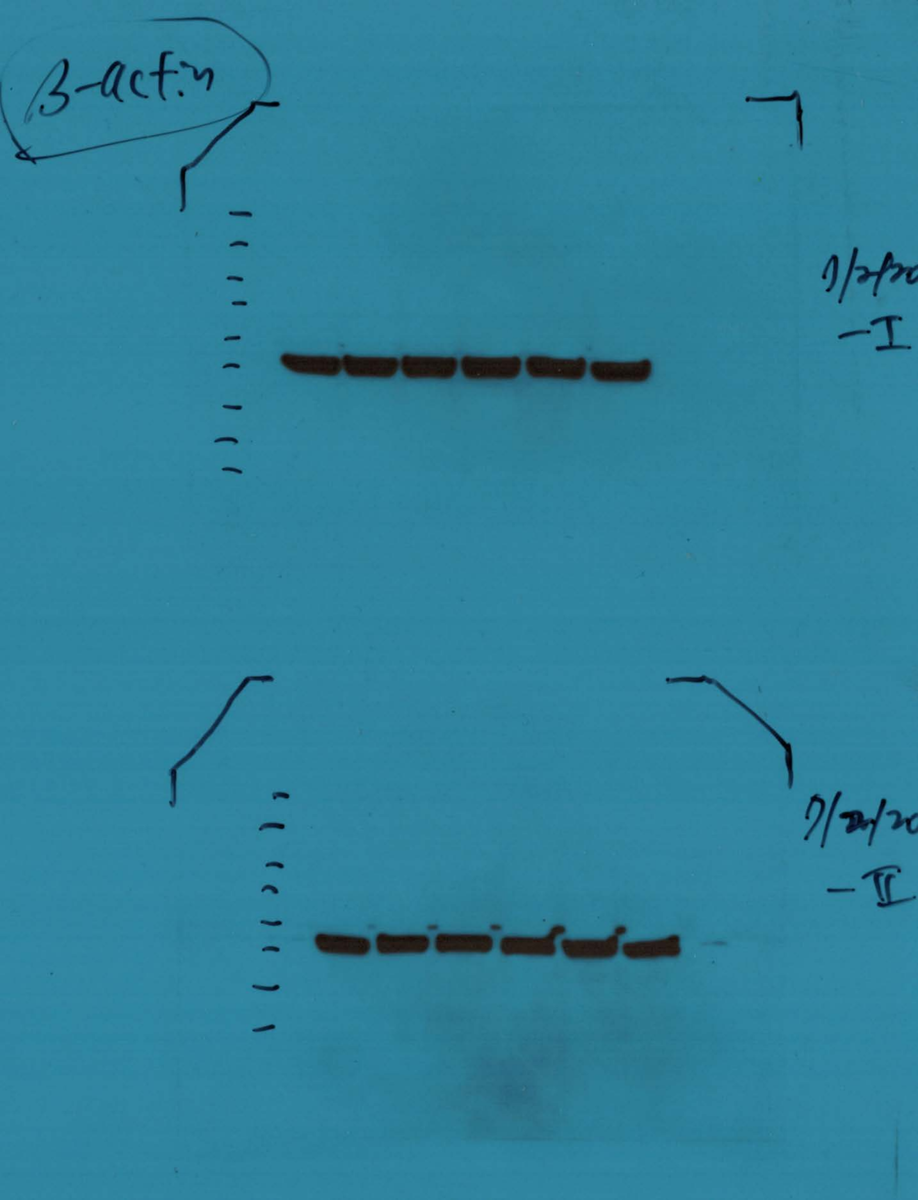
β-actin
-I
-II

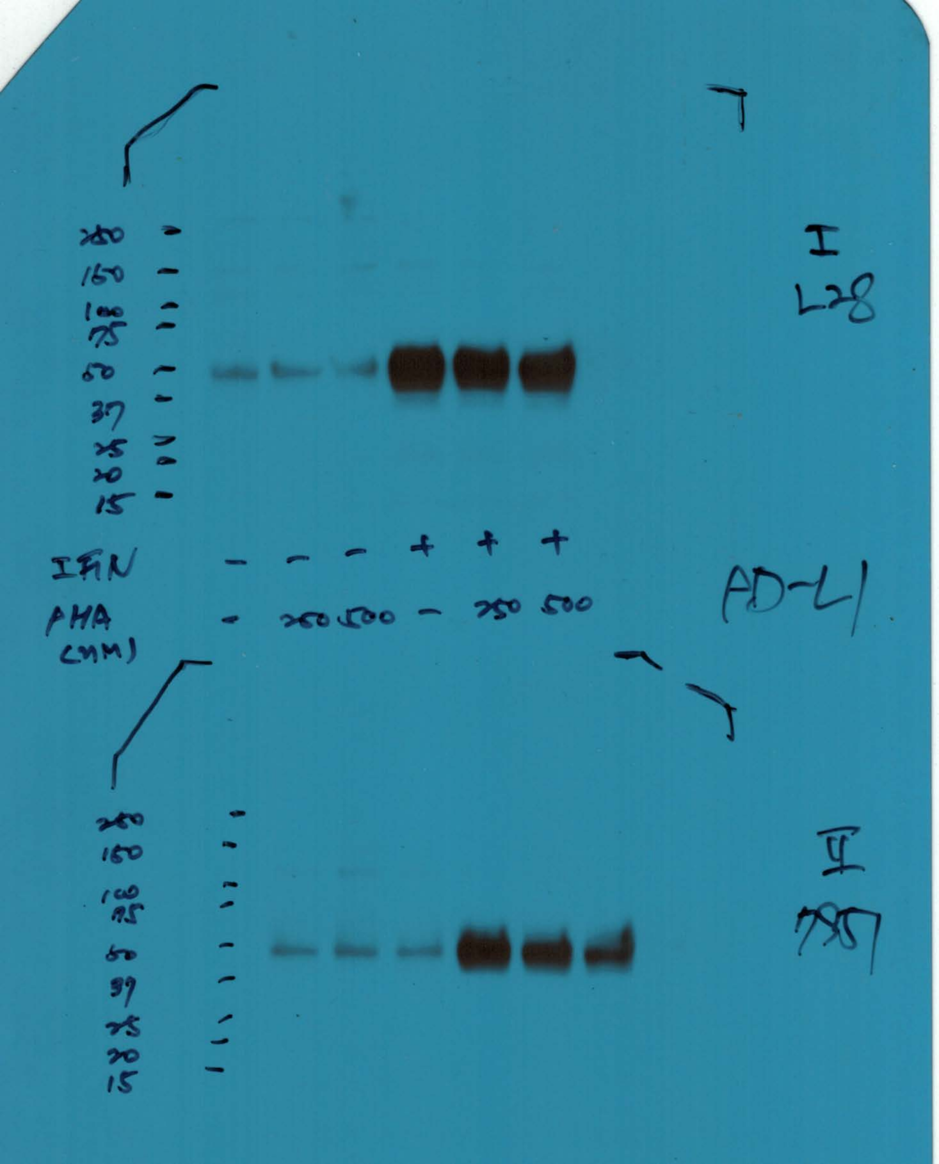

250
150
100
75
50
37
25
20
15
I
L28
IFN - - - + + +
AHA - 250 500 - 250 500
(uM)
PD-L1
250
150
100
75
50
37
25
20
15
II
