# Supplementary material for: MET Receptor Tyrosine Kinase Inhibition Reduces Interferon-Gamma (IFN-γ)-Stimulated PD-L1 Expression through the STAT3 Pathway in Melanoma Cells
Source: Cancers (Basel). 2023 Jun 29;15(13):3408. doi: 10.3390/cancers15133408 (PMC10340457; doi:10.3390/cancers15133408)
Supplement: Supplementary file 1 [file cancers-15-03408-s001.zip › Supplemental Figure S5A.pdf]

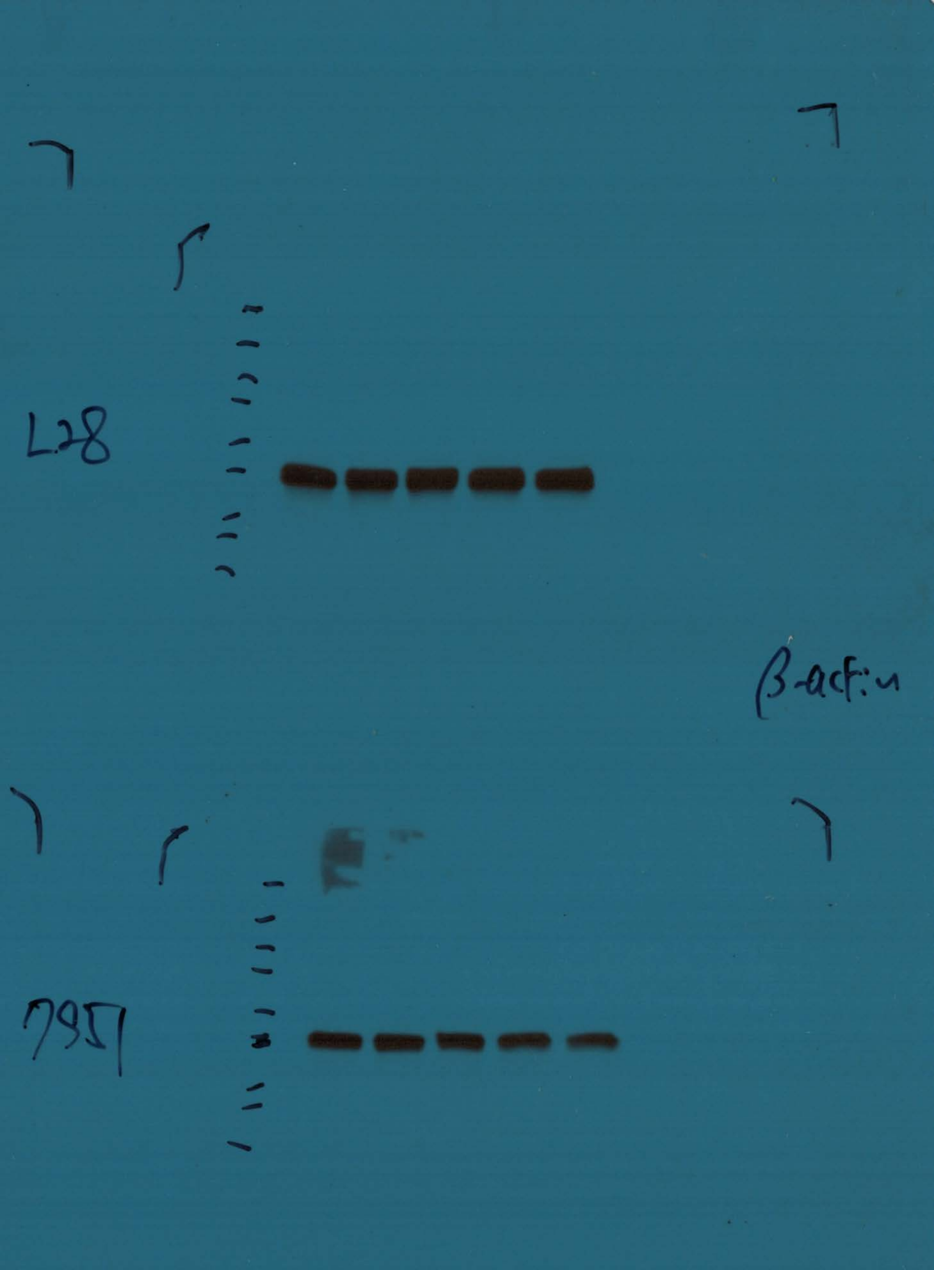
L28
β-actin

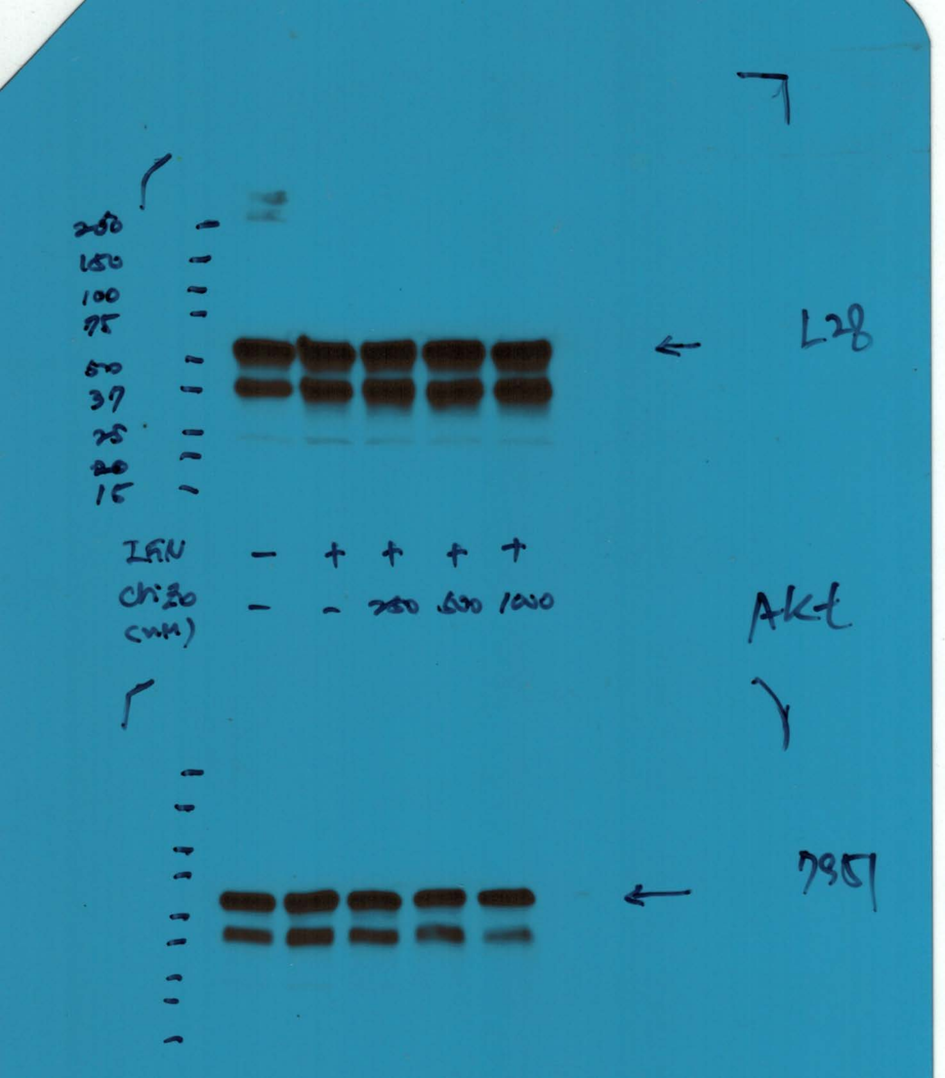
250
150
100
75
50
37
25
20
15
L28
IFN − + + + +
− − 250 500 1000
Akt

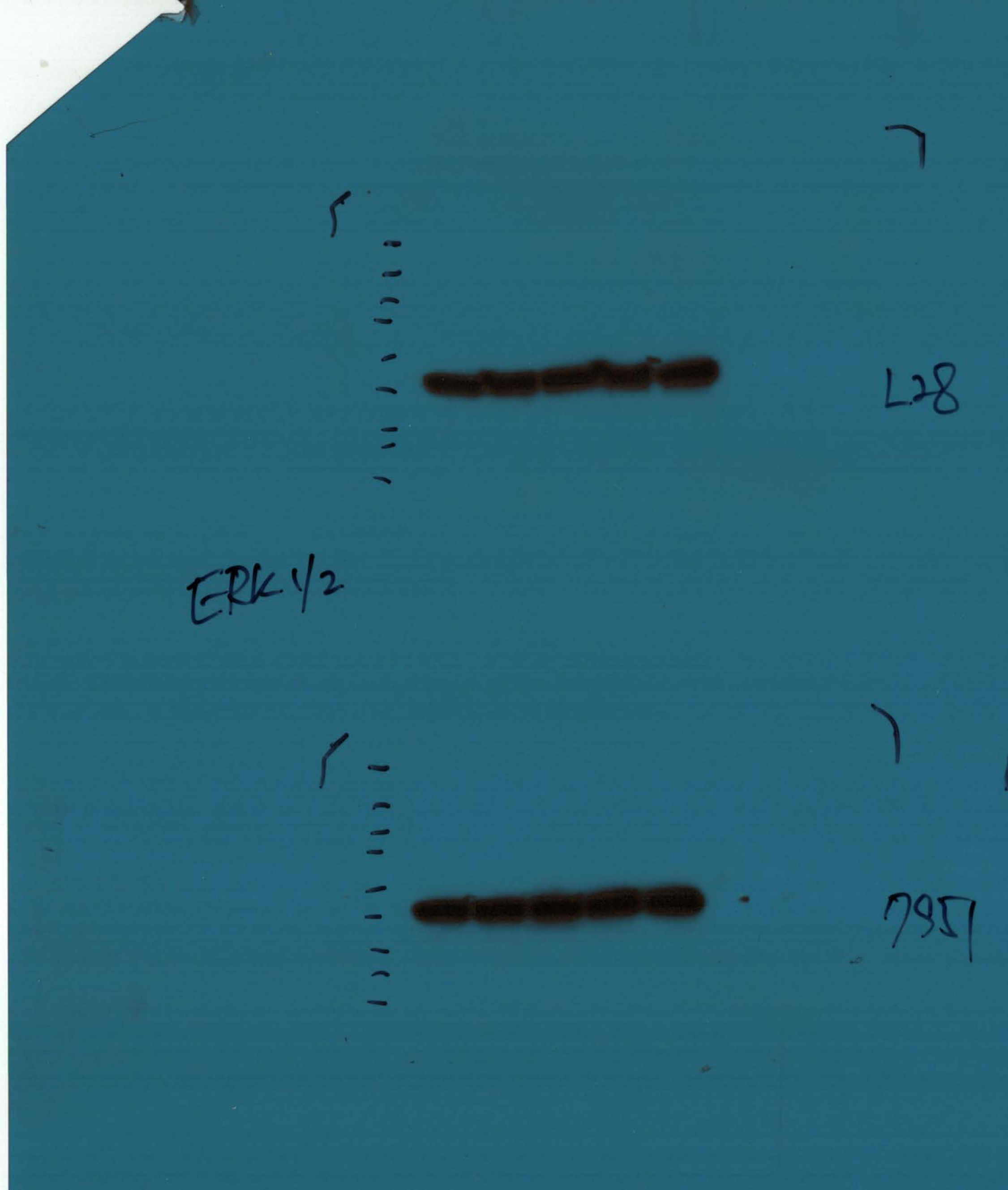
L28
ERK 1/2
7951

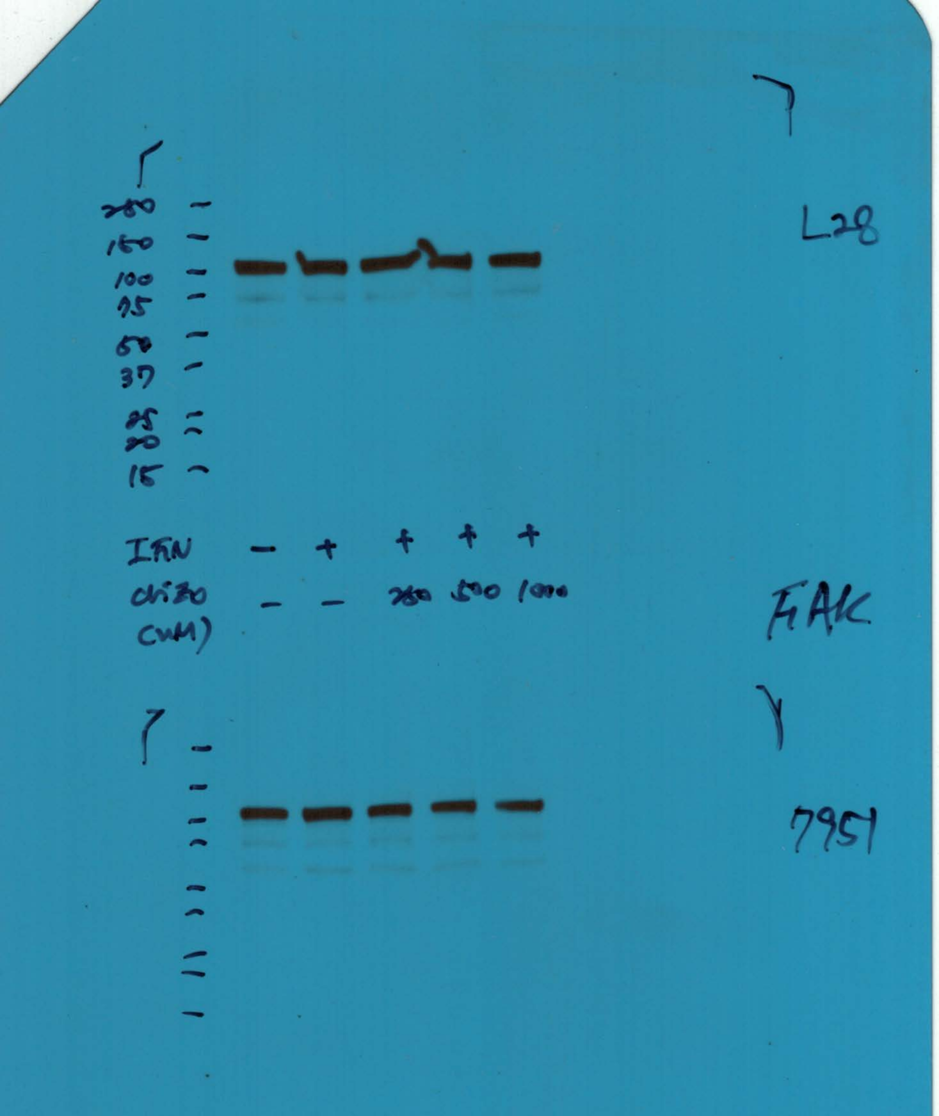

250
150
100
75
50
37
25
20
15
L28
IFN – + + + +
crizo – – 250 500 1000
(nM)
FAK
7951

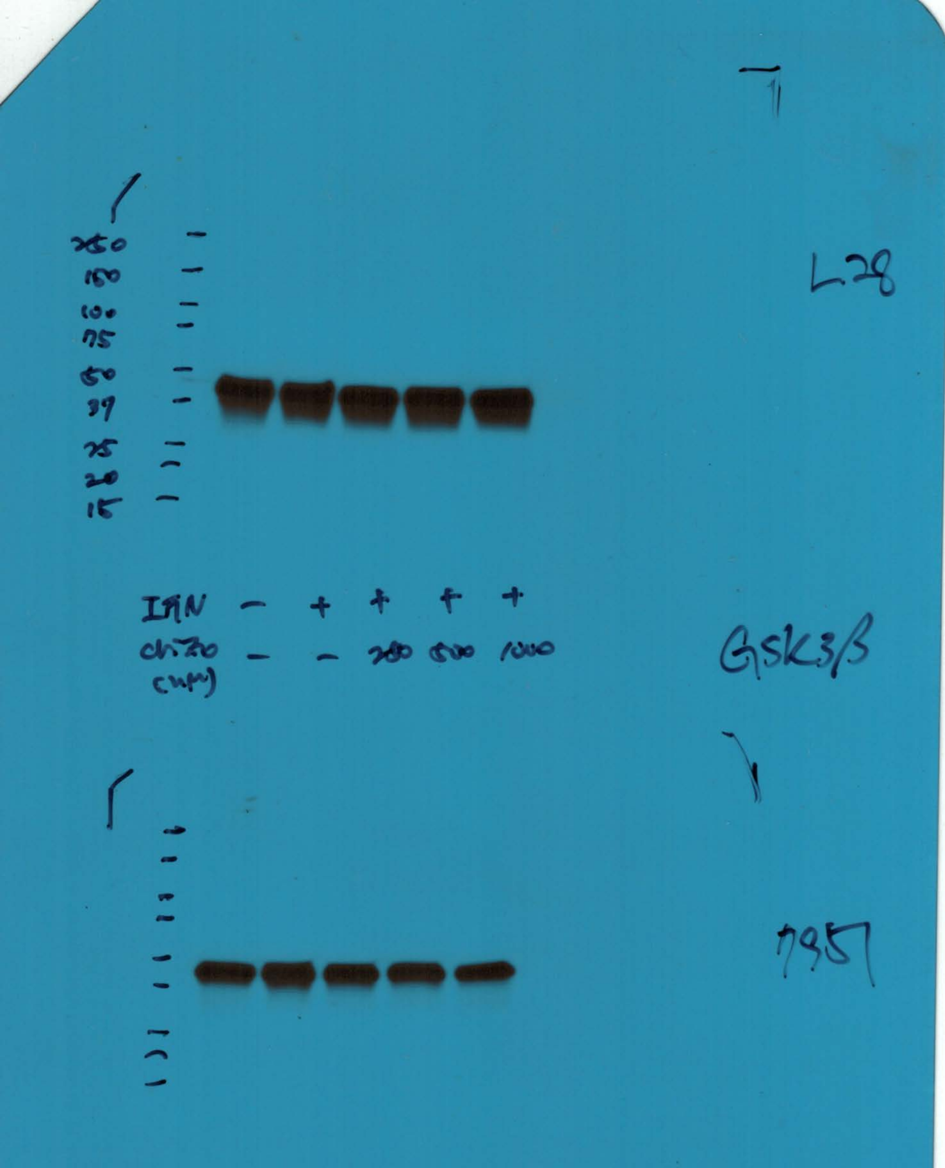

250
150
100
75
50
37
25
20
15
L28
IFN − + + + +
− − 250 1000
(nM)
GSK3β

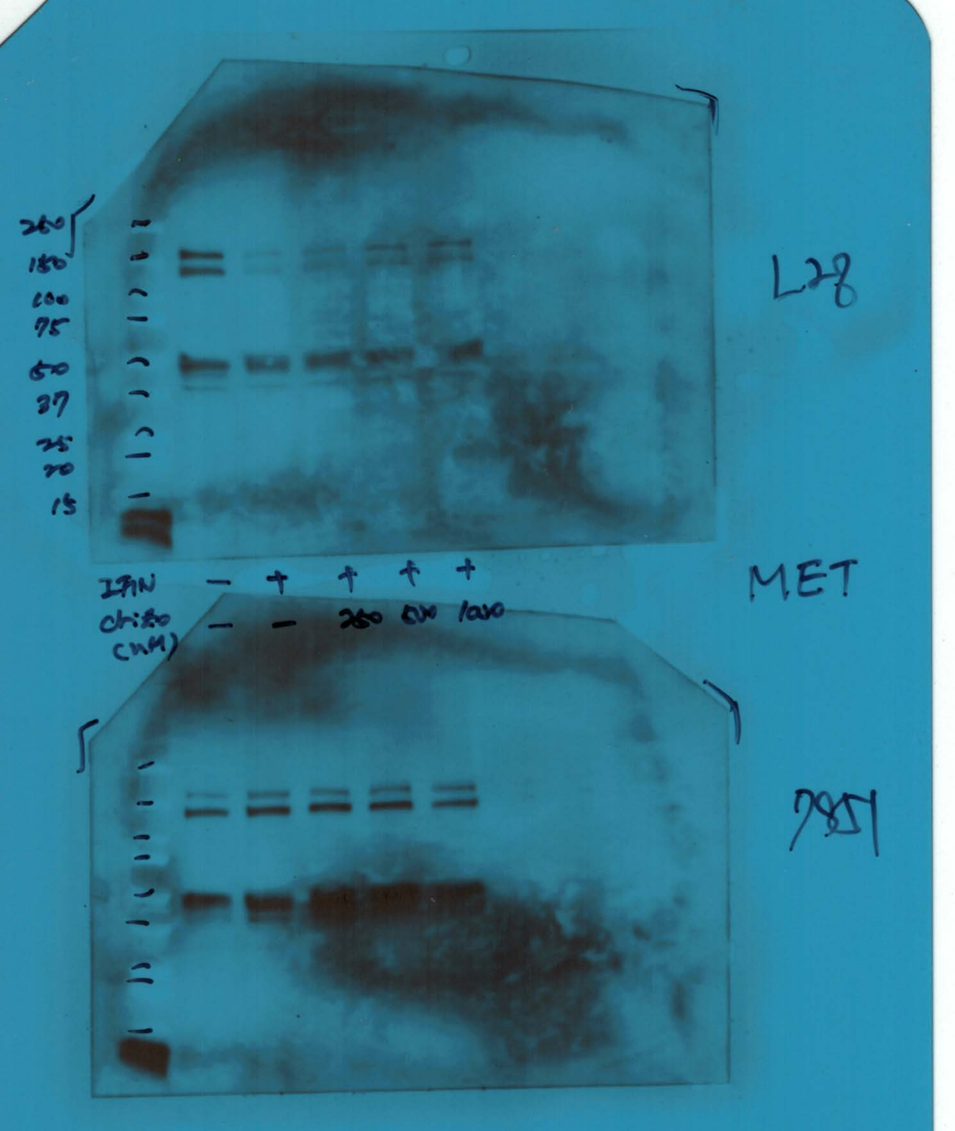
250
150
100
75
50
37
25
20
15
L28
IFN – + + + +
– – 250 500 1000
(nM)
MET
7851

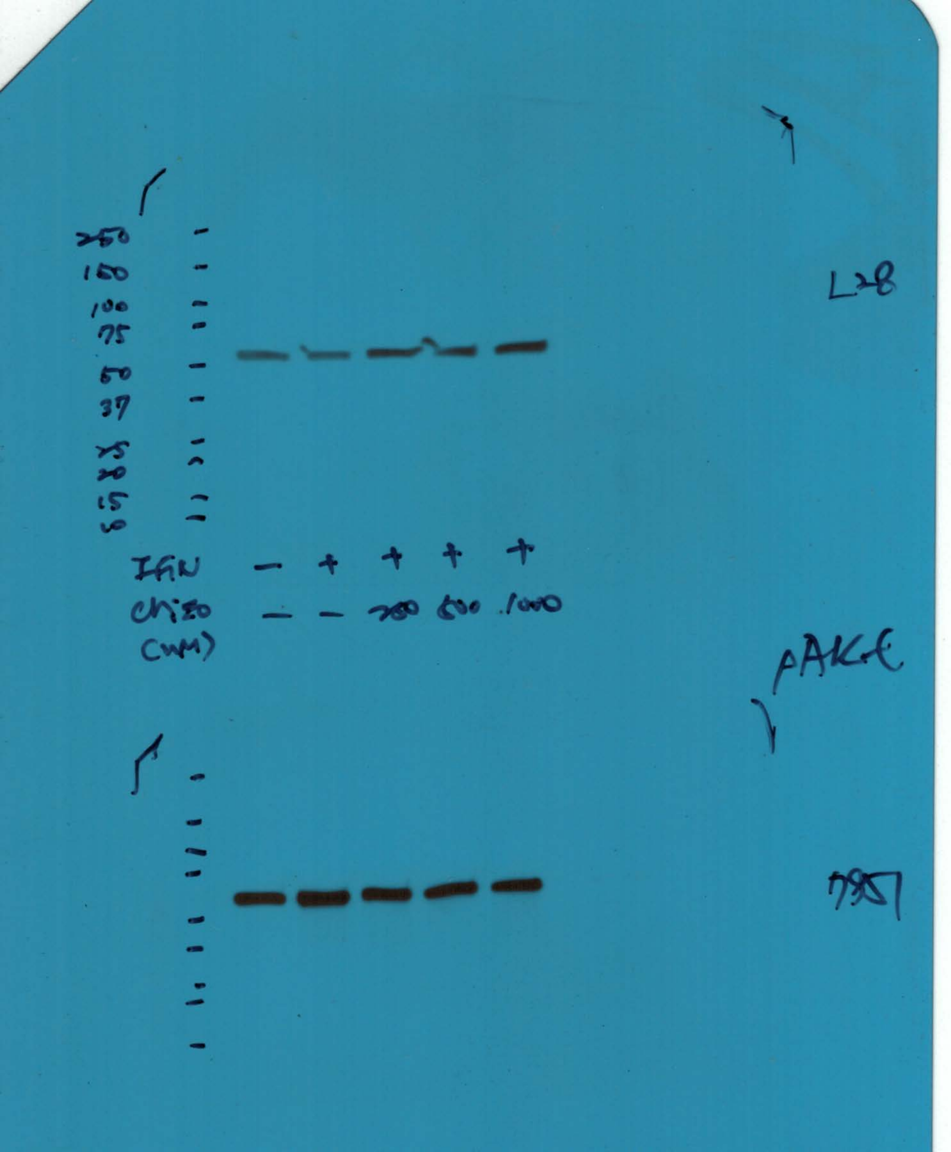
250
150
100
75
50
37
25
20
15
10
L28
IFN
— + + + +
250 500 1000
pAKt

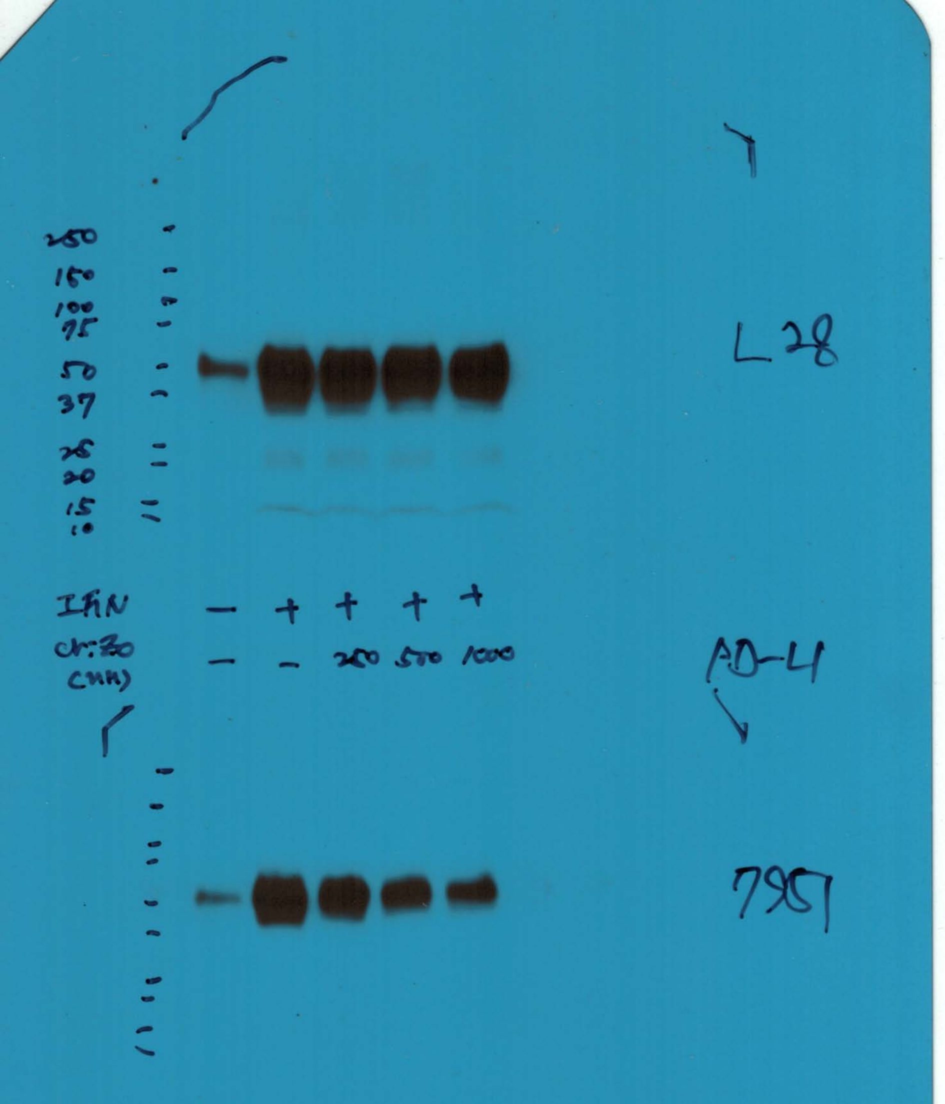
250
150
100
75
50
37
25
20
15
10
IFN
− + + + +
(nM)
− − 250 500 1000
L28
PD-L1

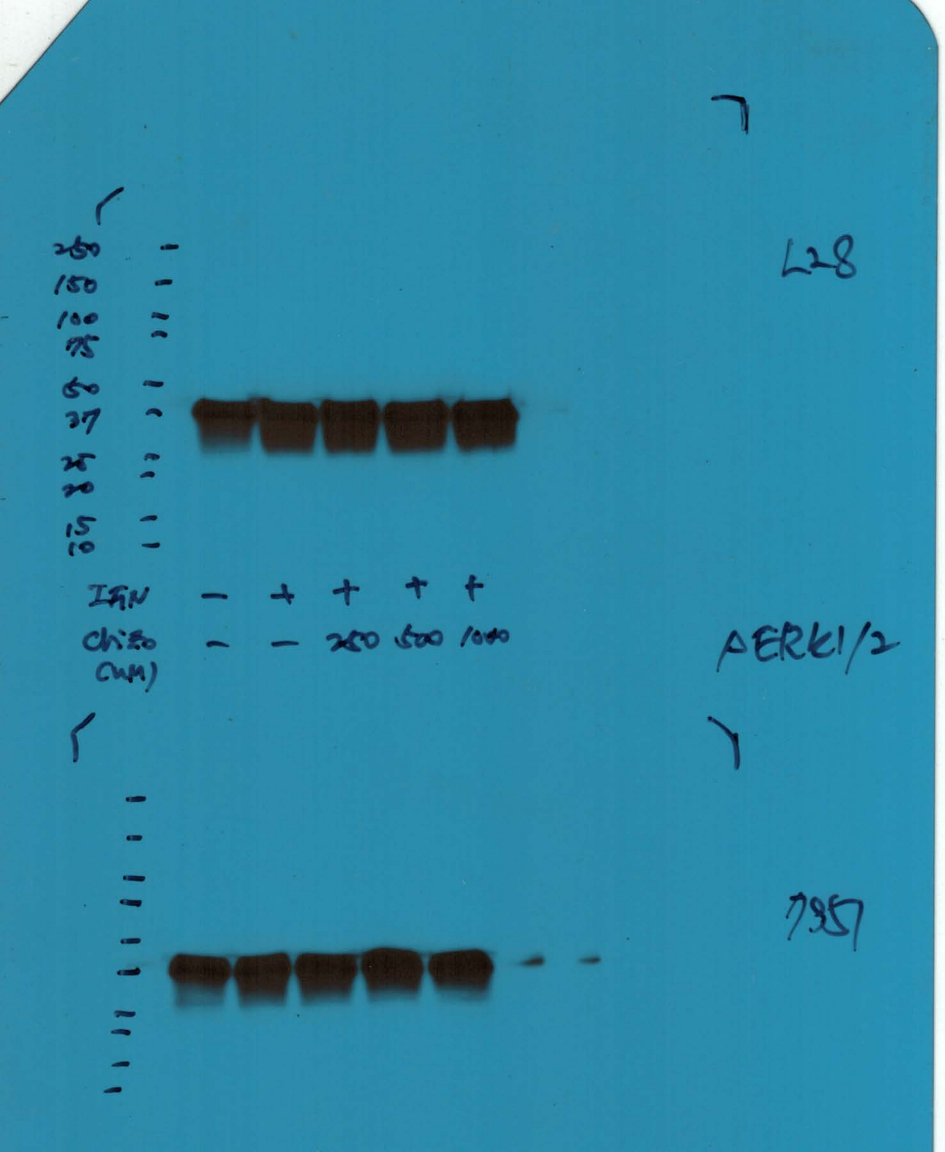

L28
250
150
100
75
50
37
25
20
15
10
IFN − + + + +
− − 250 500 1000
pERK1/2

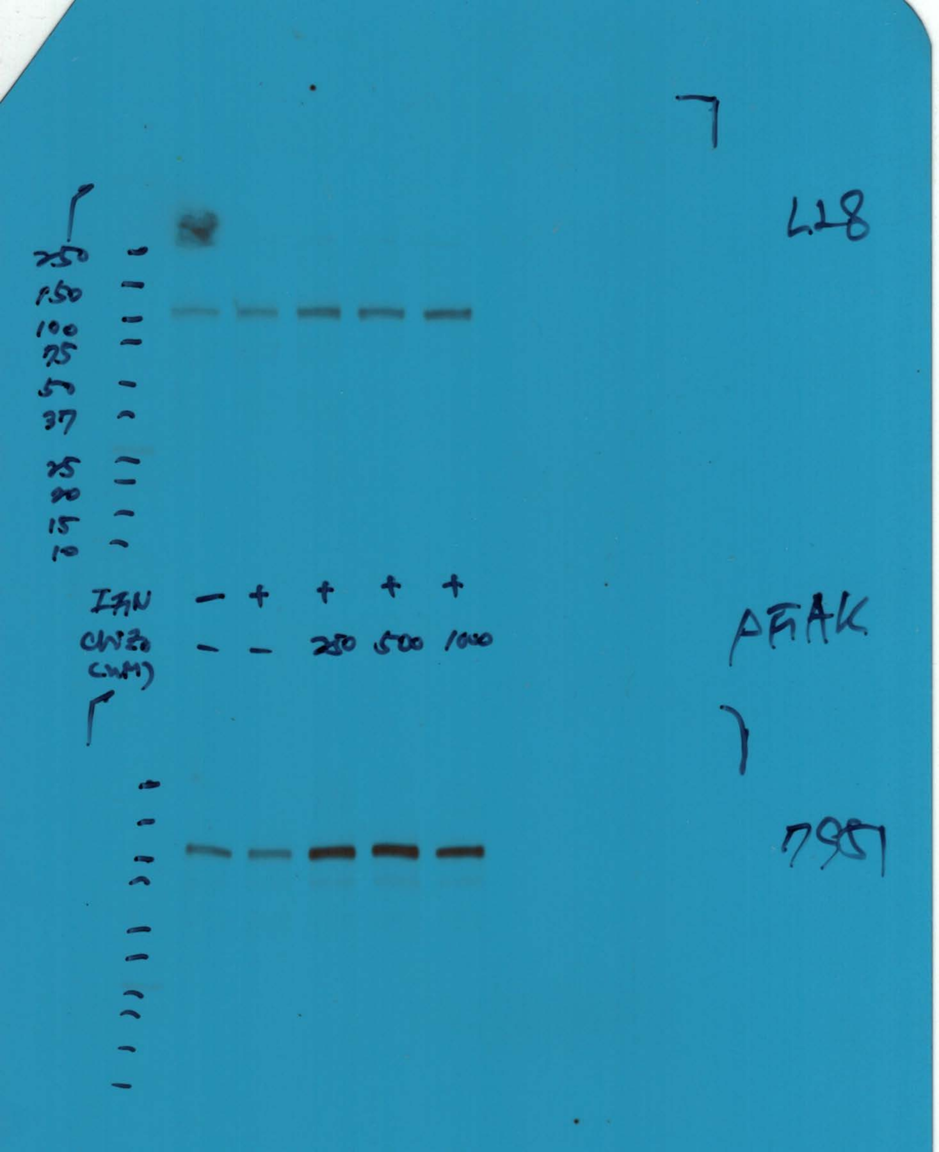
250
150
100
75
50
37
25
20
15
10
IFN
250
500
1000
(nM)

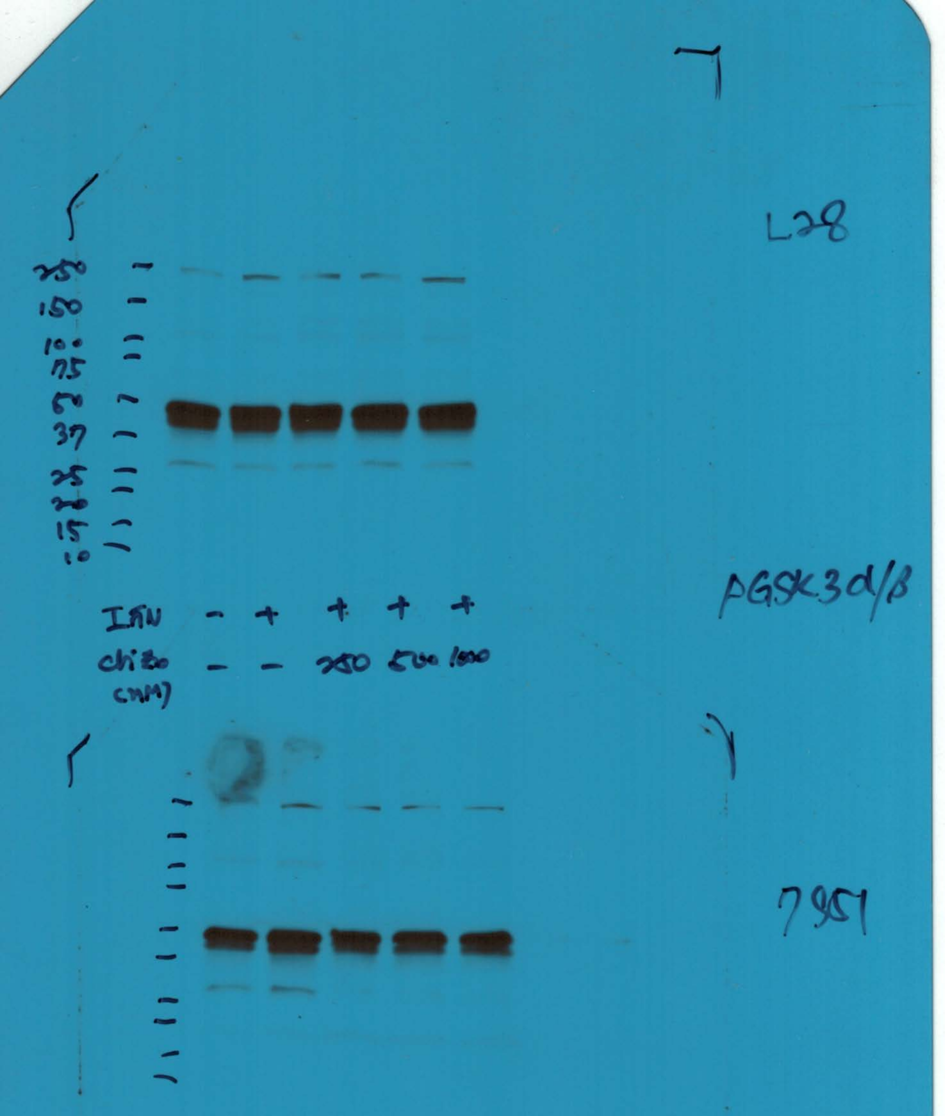

L28
250
150
100
75
50
37
25
20
15
10
pGSK3α/β
IFN - + + + +
- - 250 500 1000
(mM)
7951

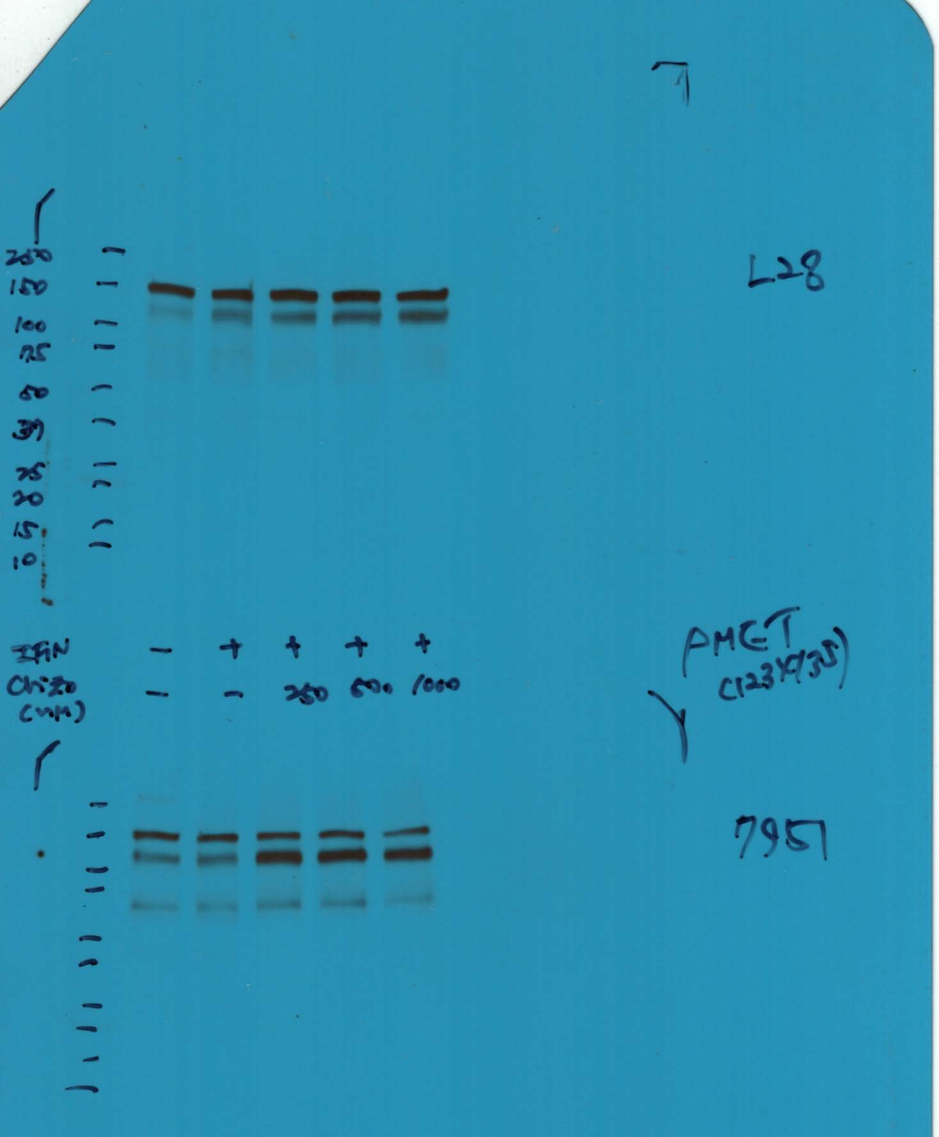
250
150
100
75
50
37
25
20
15
10
L28
IFN − + + + +
− − 250 500 1000
(nM)
PMET
7951

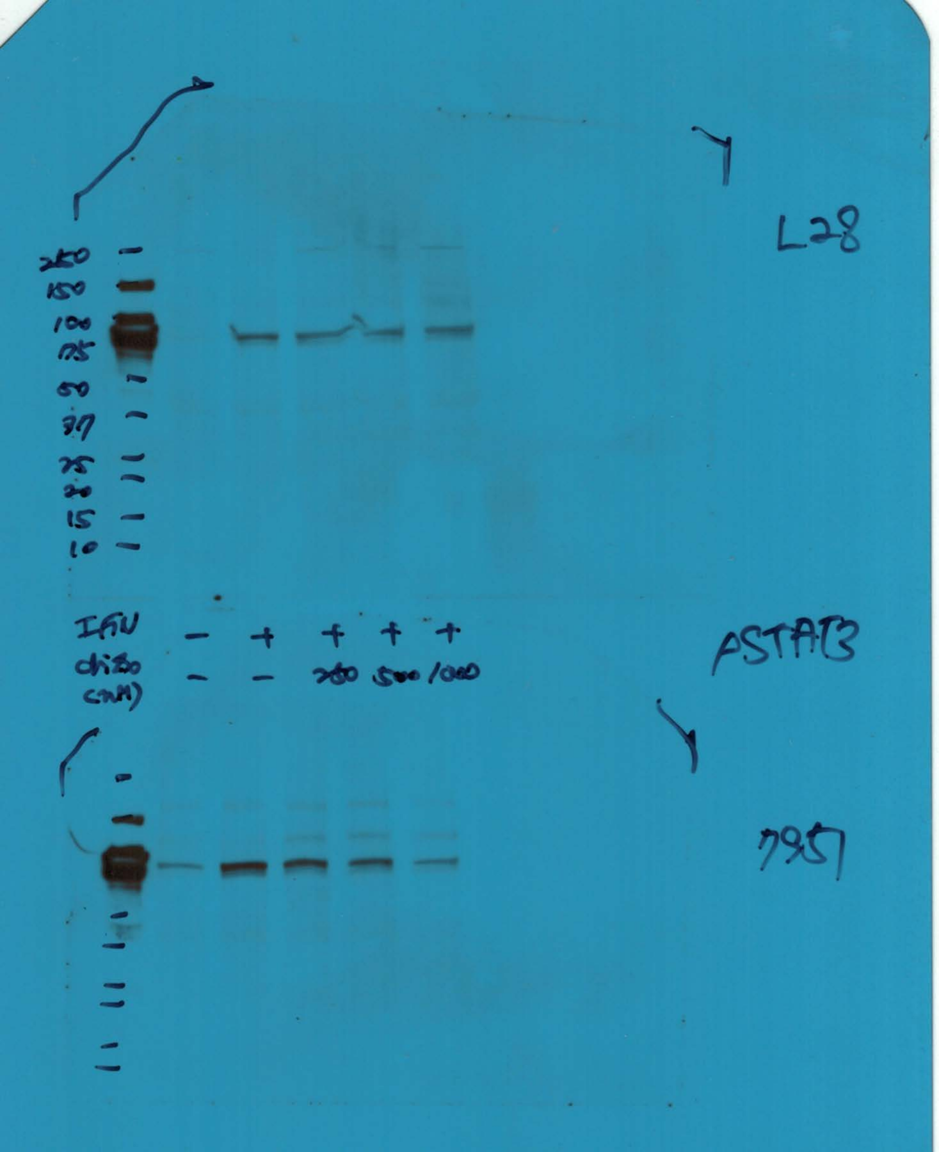
L28
250
150
100
75
50
37
25
20
15
10
IFN − + + + +
chi30 − − 250 500 1000
(nM)
pSTAT3
7957

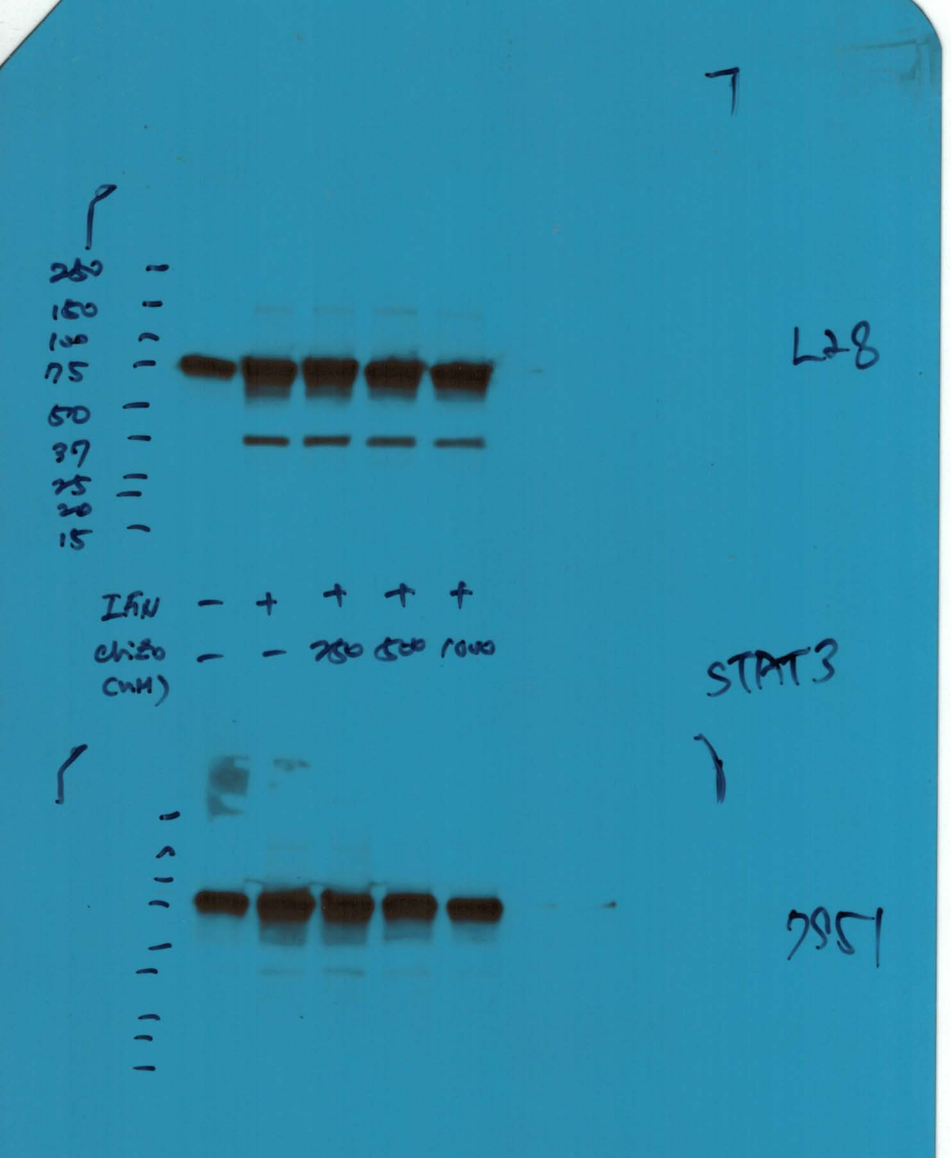
7
250
150
100
75
50
37
25
20
15
L28
IFN - + + + +
chito - - 250 500 1000
(nM)
STAT3
7851
