# Supplementary material for: MET Receptor Tyrosine Kinase Inhibition Reduces Interferon-Gamma (IFN-γ)-Stimulated PD-L1 Expression through the STAT3 Pathway in Melanoma Cells
Source: Cancers (Basel). 2023 Jun 29;15(13):3408. doi: 10.3390/cancers15133408 (PMC10340457; doi:10.3390/cancers15133408)
Supplement: Supplementary file 1 [file cancers-15-03408-s001.zip › Supplemental Figure S6A.pdf]

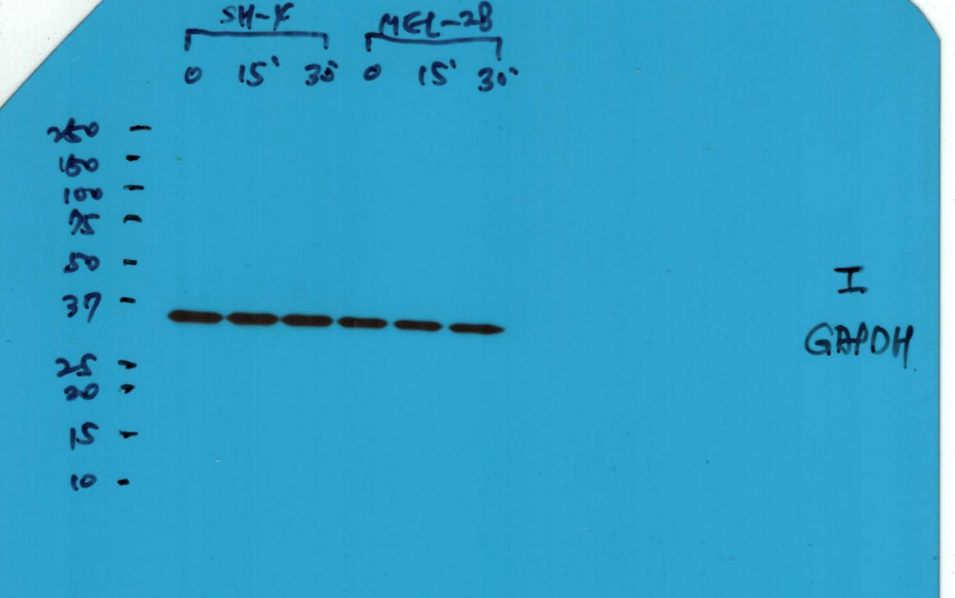

MEL-28
0 15' 30' 0 15' 30'
250
150
100
75
50
37
25
20
15
10
I
GAPDH

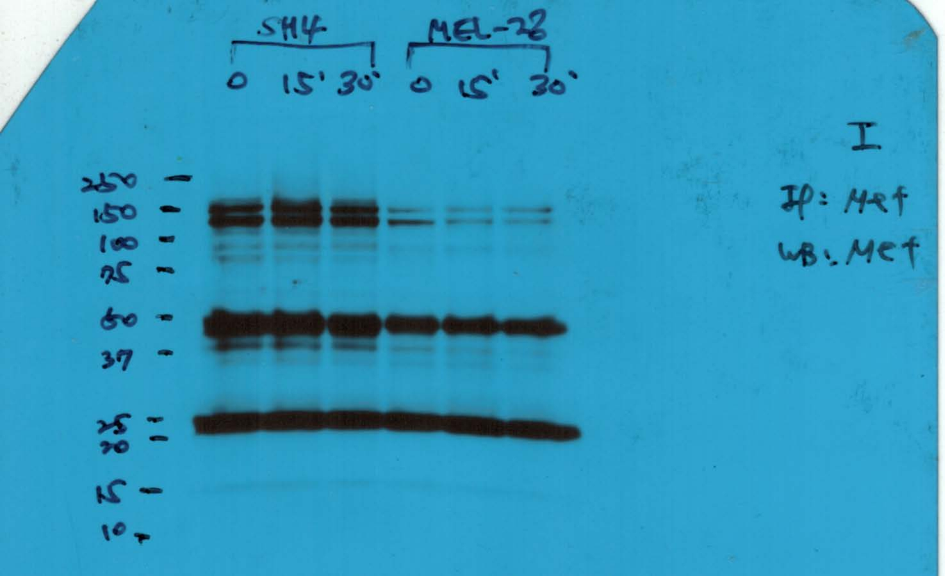
SH4
MEL-28
0
15'
30'
0
15'
30'
250
150
100
75
50
37
25
20
15
10
I
IP: Met
WB: Met

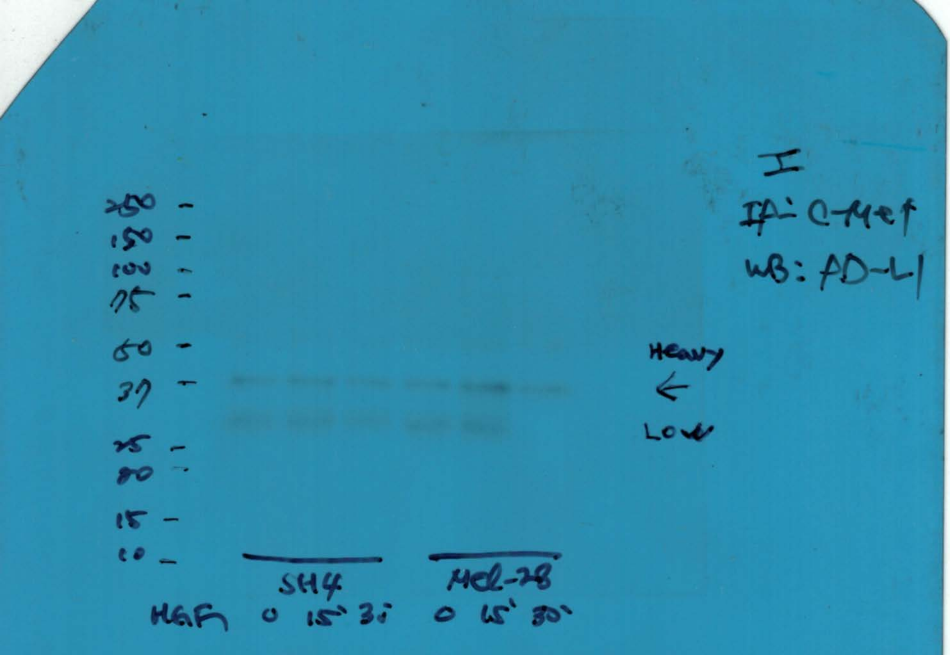
I
IP: c-Met
WB: PD-L1
250
150
100
75
50
37
25
20
15
10
Heavy
Low
SH4
Mel-28
HGF 0 15' 30' 0 15' 30'

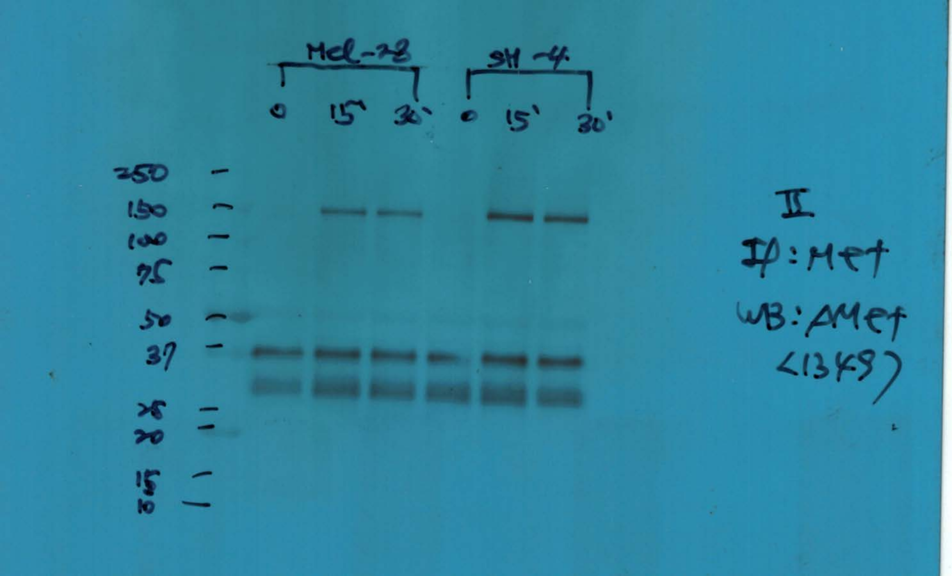
Mel-28
SH-4
0
15'
30'
0
15'
30'
250
150
100
75
50
37
25
20
15
10
II
IP: Met
WB: Met
(1349)

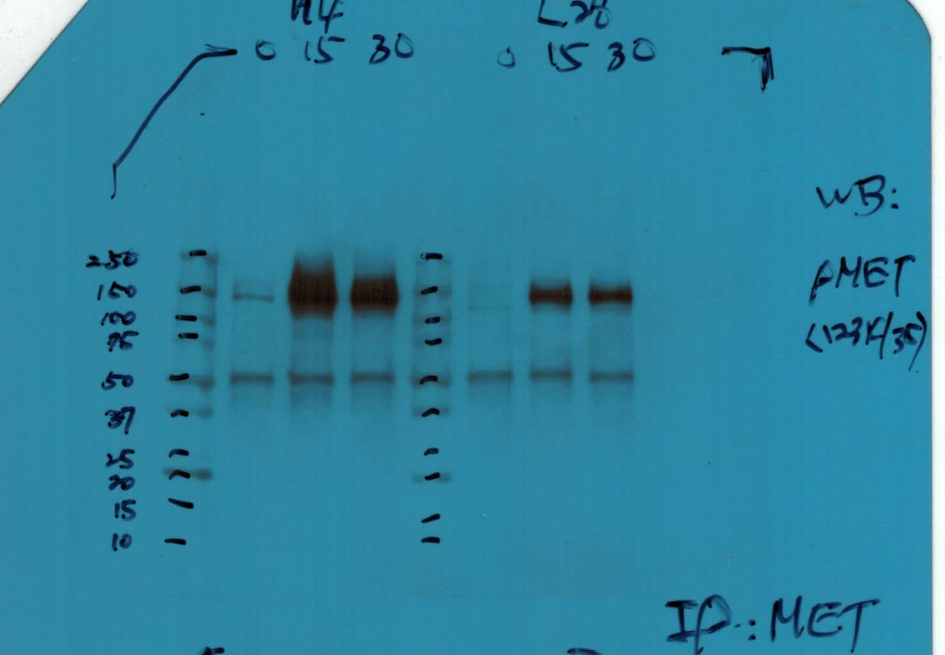
0 15 30
0 15 30
WB:
pMET
(1234/35)
IP: MET
250
150
100
75
50
37
25
20
15
10

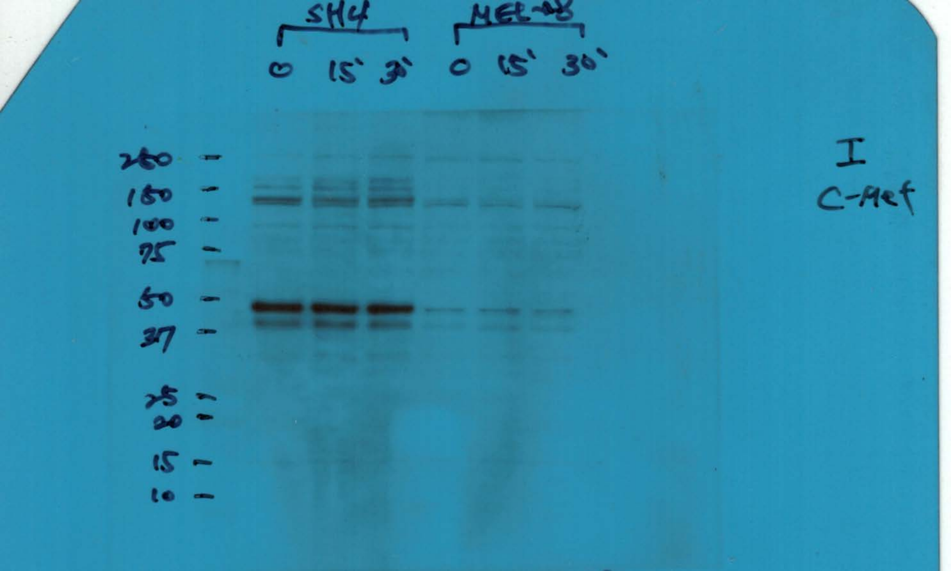
SH4
MEL-28
0 15' 30' 0 15' 30'
250
150
100
75
50
37
25
20
15
10
I
C-Met

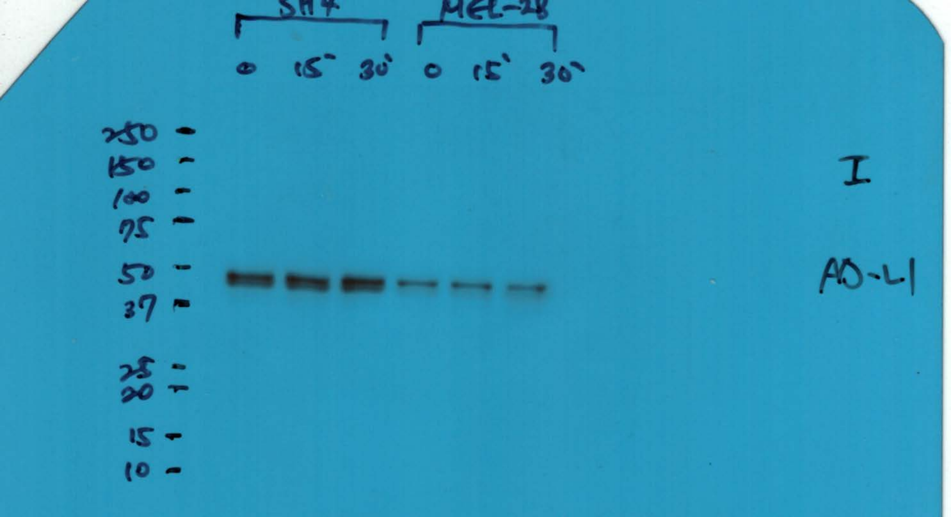
SH4
MEL-28
0 15' 30' 0 15' 30'
250
150
100
75
50
37
25
20
15
10
I
PD-L1
