# Supplementary material for: MET Receptor Tyrosine Kinase Inhibition Reduces Interferon-Gamma (IFN-γ)-Stimulated PD-L1 Expression through the STAT3 Pathway in Melanoma Cells
Source: Cancers (Basel). 2023 Jun 29;15(13):3408. doi: 10.3390/cancers15133408 (PMC10340457; doi:10.3390/cancers15133408)
Supplement: Supplementary file 1 [file cancers-15-03408-s001.zip › Supplemental Table S1.pdf]

# Supplemental Table S1

| Name | Tumor source | Tissue | STR profile | Sequence variations | Pigmentation |
|---|---|---|---|---|---|
| **WM35** | Primary | superficial spreading | Link | BRAF p.Val600Glu (c.1799T>A) | - |
| **SK-MEL-28** | Metastasis | Axillary Lymph node | Link | BRAF p.Val600Glu (c.1799T>A)<br>CDK4 p.Arg24Cys (c.70C>T)<br>EGFR p.Pro753Ser (c.2257C>T)<br>PTEN p.Thr167Ala (c.499A>G)<br>TP53 p.Leu145Arg (c.434T>G) | - |
| **RPMI-7951** | Metastasis | Lymph node | Link | BRAF p.Val600Glu (c.1799T>A)<br>PTEN deletion | - |
| **SH-4** | Metastasis | Pleural effusion | Link | BRAF p.Val600Glu (c.1799T>A) | Yes |
