# Supplementary figures and images for: MET Receptor Tyrosine Kinase Inhibition Reduces Interferon-Gamma (IFN-γ)-Stimulated PD-L1 Expression through the STAT3 Pathway in Melanoma Cells
Source: Cancers (Basel). 2023 Jun 29;15(13):3408. doi: 10.3390/cancers15133408 (PMC10340457; doi:10.3390/cancers15133408)

Supplemental Figure S2

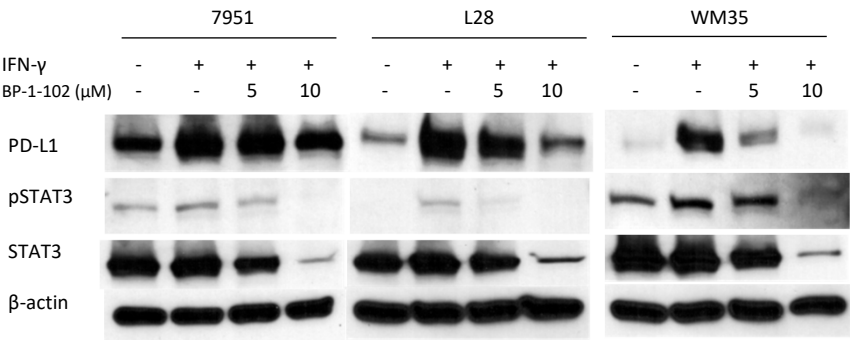

Supplement: Supplementary file 1 [file cancers-15-03408-s001.zip › Supplemental Figure S2.pdf]

Supplemental Figure S3

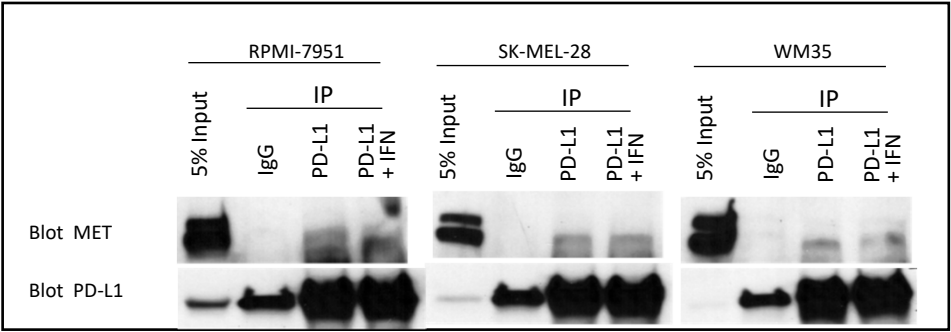

Supplement: Supplementary file 1 [file cancers-15-03408-s001.zip › Supplemental Figure S3.pdf]

| SHK |     |     | MEL-28 |     |     |
|-----|-----|-----|--------|-----|-----|
| 0   | 15' | 30' | 0      | 15' | 30' |

250 -  
 150 -  
 100 -  
 75 -  
 50 -  
 37 -  
 25 -  
 20 -  
 15 -

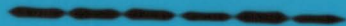

GRPDH

SH-4      MEL-28  
 0   15'   30'      0   15'   30'

II

IP: PD-L1

Blot: C-Met

250  
150  
100  
75  
50  
37  
25  
20  
15  
10

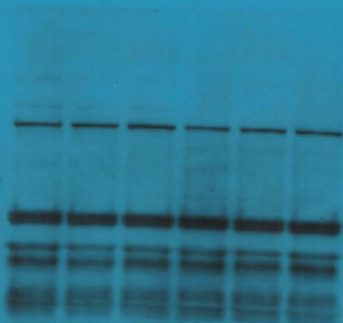

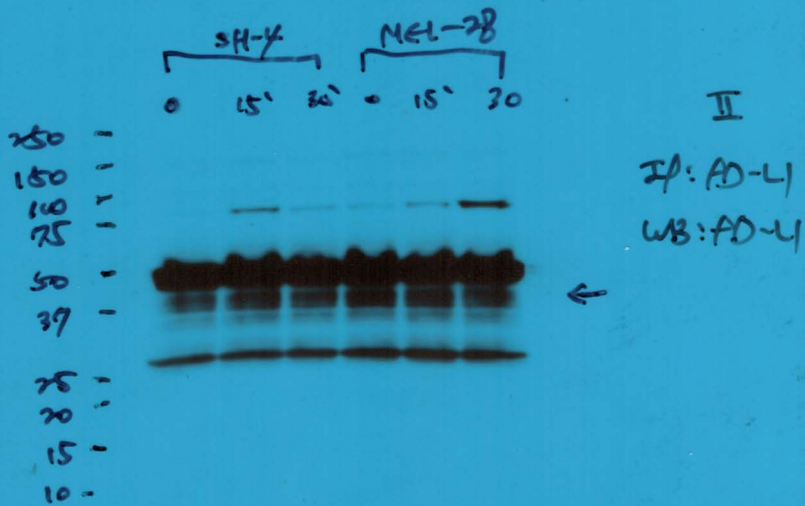

H4

L28

0 15 30

0 15 30

IP: AD-4

WB: PMET  
(123K/35)

250  
150  
100  
75  
50  
37  
25  
20  
15  
10

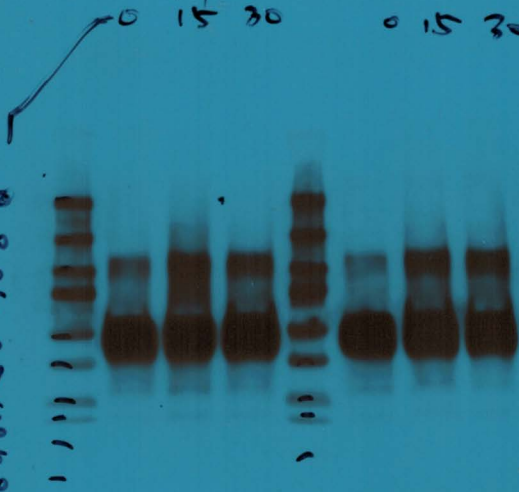

HY

L28

0 15 30

0 15 30

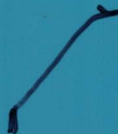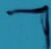

IP: ADL4

PMET  
(13K9)

250  
150  
100  
75  
50  
37  
25  
20  
15  
10

1 1 1 1 1 1 1 1 1 1

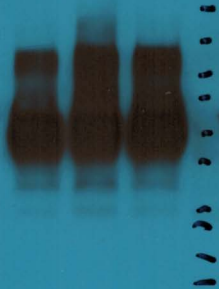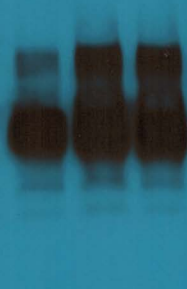

SH-4      MEL-28  
 0   15' 30'      0   15' 20'

250 -  
 150 -  
 100 -  
 75 -  
 50 -  
 37 -  
 25 -  
 20 -  
 15 -  
 10 -

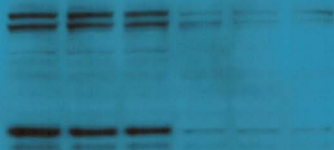

II  
 C-Met

| SH-4 |     |     | MEL-28 |     |     |
|------|-----|-----|--------|-----|-----|
| 0    | 15' | 30' | 0      | 15' | 30' |

250 -  
 150 -  
 100 -  
 75 -  
 50 -  
 37 -  
 25 -  
 20 -  
 15 -

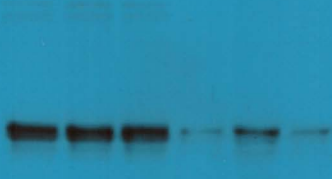

I

AD-4

Supplement: Supplementary file 1 [file cancers-15-03408-s001.zip › Supplemental Figure S6B.pdf]

| SHK |     |     | MEL-28 |     |     |
|-----|-----|-----|--------|-----|-----|
| 0   | 15' | 30' | 0      | 15' | 30' |

250 -  
 150 -  
 100 -  
 75 -  
 50 -  
 37 -  
 25 -  
 20 -  
 15 -

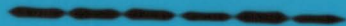

GRPDH

SH-4      MEL-28  
 0 15' 30'    0 15' 30'

II

IP: PD-L1

Blot: C-Met

250  
150  
100  
75  
50  
37  
25  
20  
15  
10

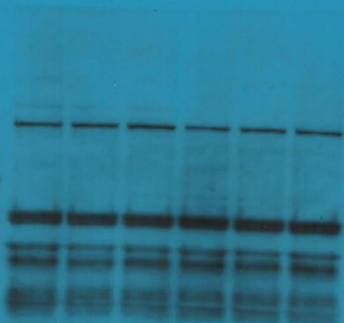

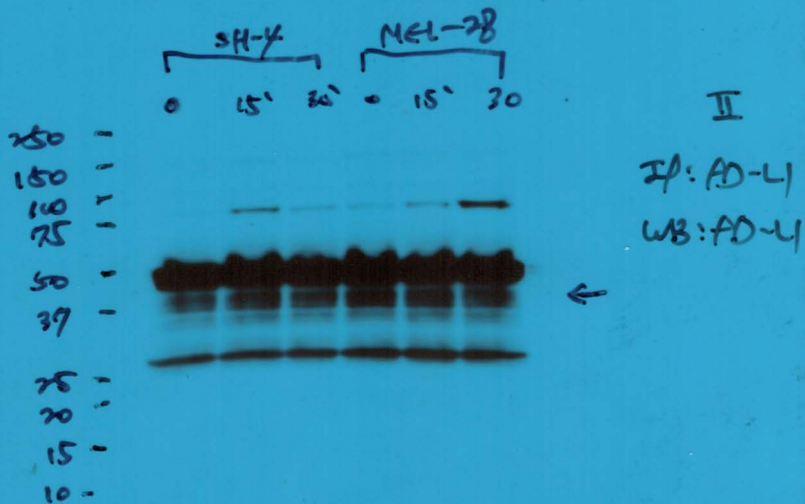

H4

L28

0 15 30

0 15 30

IP: AD-4

WB: PMET  
(123K/35)

250  
150  
100  
75  
50  
37  
25  
20  
15  
10

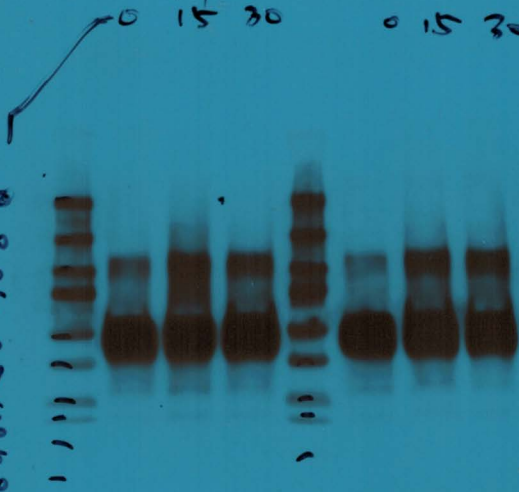

HY

L28

0 15 30

0 15 30

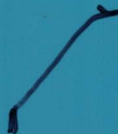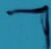

IP: ADL4

PMET  
(13K9)

250  
150  
100  
75  
50  
37  
25  
20  
15  
10

1 1 1 1 1 1 1 1 1 1

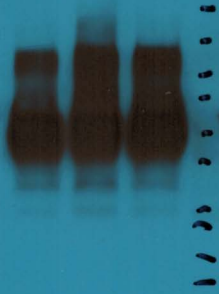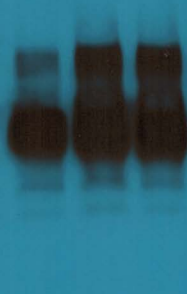

SH-4      MEL-28  
 0   15' 30'      0   15' 20'

250 -  
 150 -  
 100 -  
 75 -  
 50 -  
 37 -  
 25 -  
 20 -  
 15 -  
 10 -

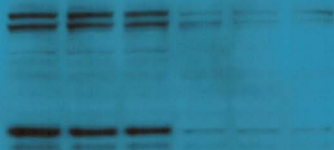

II  
 C-Met

| SH-4 |     |     | MEL-28 |     |     |
|------|-----|-----|--------|-----|-----|
| 0    | 15' | 30' | 0      | 15' | 30' |

250 -  
 150 -  
 100 -  
 75 -  
 50 -  
 39 -  
 25 -  
 20 -  
 5 -

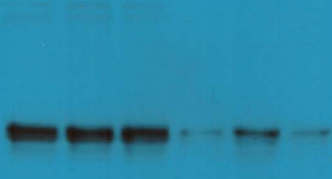

I

AD-4

Supplement: Supplementary file 1 [file cancers-15-03408-s001.zip › Supplemental Figure S6C.pdf]

7957

35

0 15 30

0 15 30

250  
150  
100  
75  
50  
37  
25  
15  
10

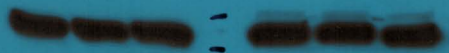

WB.

$\beta$ -actin

7957

15' 30"

35

15' 30"

IP: PD-4

I

MET

280  
150  
100  
75  
50  
39  
25  
20  
15

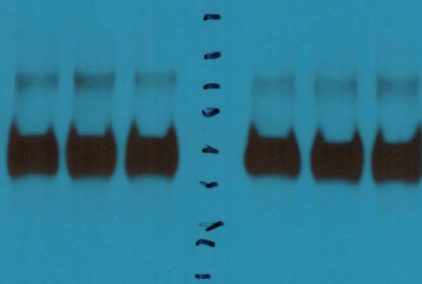

IP: ADL

7957

0 15' 30'

35

0 15' 30'

200  
150  
100  
95  
60  
39  
25  
20  
15  
10

WB.

ADL

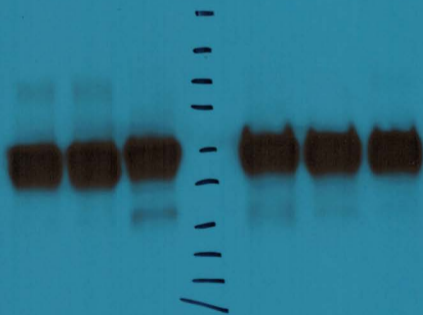

7857  
0 15' 30'

35  
0 15' 30'

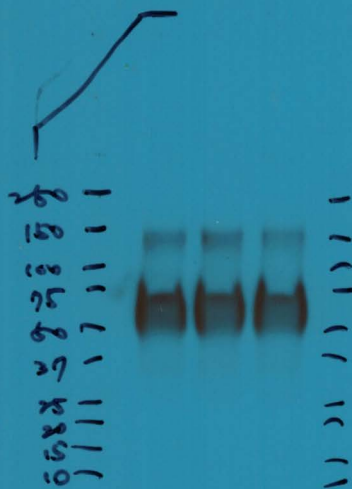

I  
AMET  
(123/11)

7857  
0 15' 30'

35  
0 15' 30'

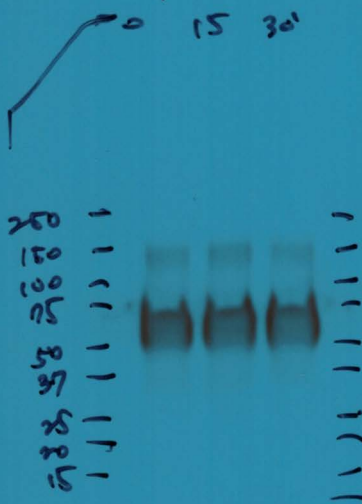

II = PD-4

II  
AMET  
(123/8)

17987

35

0 15' 30'

0 15' 30'

7

250  
150  
100  
75  
50  
37  
25  
20  
15  
0

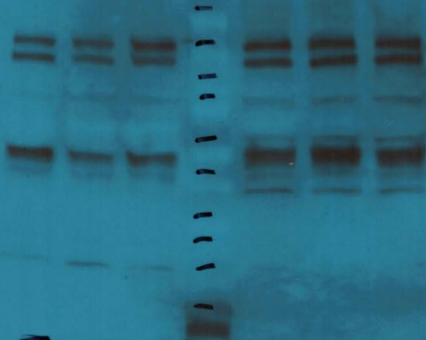

WB.  
MET

17987

35

0 15' 30'

0 15' 30'

250  
150  
100  
75  
50  
37  
25  
20  
15  
0

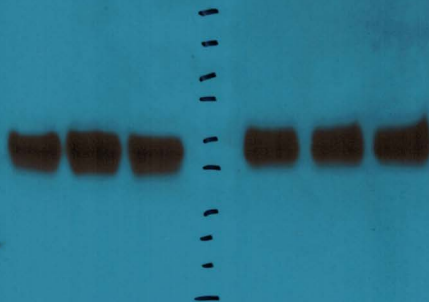

WB  
ADL1

Supplement: Supplementary file 1 [file cancers-15-03408-s001.zip › Supplemental Figure S6D.pdf]
